# A DIO2 missense mutation and its impact on fetal response to PRRSV infection

**DOI:** 10.1186/s12917-024-04099-4

**Published:** 2024-06-12

**Authors:** Haesu Ko, J. Alex Pasternak, Margaret K. Mulligan, Glenn Hamonic, Naresh Ramesh, Daniel J. MacPhee, Graham S. Plastow, John C. S. Harding

**Affiliations:** 1 Western College of Veterinary Medicine, University of Saskatchewan, Saskatoon, SK S7N5B4 Canada; 2 Department of Agricultural, Food and Nutritional Science, University of Alberta, Edmonton, AB T6G2H1 Canada; 3 Department of Animal Sciences, Purdue University, West Lafayette, IN 47907 USA; 4 Department of Biology, West Virginia University Institute of Technology, Beckley, WV 25801 USA

**Keywords:** PRRSV, Swine, Fetus, Thyroid, Deiodinase, Nonsynonymous mutation

## Abstract

**Background:**

Porcine reproductive and respiratory syndrome virus 2 (PRRSV-2) infection during late gestation substantially lowers fetal viability and survival. In a previous genome-wide association study, a single nucleotide polymorphism on chromosome 7 was significantly associated with probability of fetuses being viable in response to maternal PRRSV-2 infection at 21 days post maternal inoculation. The *iodothyronine deiodinase 2* (*DIO2*) gene, located ~ 14 Kilobase downstream of this SNP, was selected as a priority candidate related to fetal susceptibility following maternal PRRSV-2 infection. Our objectives were to identify mutation(s) within the porcine *DIO2* gene and to determine if they were associated with fetal outcomes after PRRSV-2 challenge. Sequencing of the *DIO2*, genotyping identified variants, and association of *DIO2* genotypes with fetal phenotypes including *DIO2* mRNA levels, viability, survival, viral loads, cortisol and thyroid hormone levels, and growth measurements were conducted.

**Results:**

A missense variant (p.Asn91Ser) was identified in the parental populations from two independent PRRSV-2 challenge trials. This variant was further genotyped to determine association with fetal PRRS outcomes. *DIO2* mRNA levels in fetal heart and kidney differed by the genotypes of Asn91Ser substitution with significantly greater *DIO2* mRNA expression in heterozygotes compared with wild-type homozygotes (*P* < 0.001 for heart, *P* = 0.002 for kidney). While Asn91Ser did not significantly alter fetal viability and growth measurements, interaction effects of the variant with fetal sex or trial were identified for fetal viability or crown rump length, respectively. However, this mutation was not related to dysregulation of the hypothalamic-pituitary-adrenal and thyroid axis, indicated by no differences in circulating cortisol, T4, and T3 levels in fetuses of the opposing genotypes following PRRSV-2 infection.

**Conclusions:**

The present study suggests that a complex relationship among *DIO2* genotype, *DIO2* expression, fetal sex, and fetal viability may exist during the course of fetal PRRSV infection. Our study also proposes the increase in cortisol levels, indicative of fetal stress response, may lead to fetal complications, such as fetal compromise, fetal death, or premature farrowing, during PRRSV infection.

**Supplementary Information:**

The online version contains supplementary material available at 10.1186/s12917-024-04099-4.

## Background

Porcine reproductive and respiratory syndrome virus (PRRSV) is subclassified into two species, *Betaarterivirus suid 1* (PRRSV-1) and *Betaarterivirus suid 2* (PRRSV-2) [1], and has been circulating for over three decades in commercial swine farms worldwide. It poses a substantial and ongoing economic challenge and swine health risk. The reproductive effect of PRRSV infection during late gestation includes increased abortions, stillbirths, number of live but weak born piglets, and preweaning mortality [2, 3].

Genetic variation in fetal pigs may influence the adaptive response to *in utero* insults such as maternal malnutrition [4] or shifts in fetal endocrine and amino acid status [5]. Fetal genotype has also been shown to differentiate between resilient or susceptible phenotypes following maternal PRRSV infection during late gestation [6]. In our previous genome wide association study (GWAS), an intergenic single nucleotide polymorphism (SNP) (DRGA0008048; hereafter DRGA), which is a single nucleotide change in the DNA sequence between coding genes, was significantly associated with fetal viability in response to maternal PRRSV-2 infection [6]. The DRGA SNP explained 34.6% of the total genetic variance, suggesting the genetic variability captured by the DRGA SNP or variants in linkage disequilibrium (LD) with the DRGA SNP may drive the association with fetal viability [6]. If in strong LD then the DRGA SNP is a very good proxy or marker for the unknown causative variant(s). The favorable B allele of this SNP was associated with higher probability of fetuses being viable in response to PRRSV-2 challenge in pregnant gilts at gestation day 85 (of 115-day normal term). The DRGA SNP is located ~ 14 Kilobase (kb) downstream of the *iodothyronine deiodinase 2* (*DIO2*) gene on *Sus scrofa* chromosome 7 (SSC7). The gene of interest codes for the type II iodothyronine deiodinase, a selenoenzyme containing a selenocysteine (Sec) residue at the active site, which catalyzes the deiodination of iodothyronine substrates at the 5’ or 3’ position of the phenolic (outer) ring [7]. Iodothyronine deiodination via DIO2 encompasses the conversion of thyroxine (3,5,3′,5′-tetraiodothyronine or T4) to its more biologically active 3,5,3′-triiodothyronine (T3) as well as 3,3′,5′-triiodothyronine (rT3) to 3,3′-diiodothyronine (3,3′-T2), where human DIO2 has been shown to have a higher affinity for T4 than rT3 [8]. Moreover, conversion of T4 to T3 by DIO2 was shown to be more efficient than by iodothyronine deiodinase 1 (DIO1), another outer and inner ring deiodinase [9]. Overall, DIO2 is an important contributor to the modulation of intracellular T3 homeostasis [7]. In addition to *DIO2*, the DRGA SNP is also located ~ 68.7 kb upstream of the equally relevant *thyroid stimulating hormone receptor* (*TSHR*) gene. This receptor is constitutively expressed by follicular cells of the thyroid gland, where it mediates the response to thyroid stimulating hormone (TSH) released from the anterior pituitary, upregulating the production and release of thyroid hormone [10]. This receptor has also been found to be expressed by immune cells in peripheral lymphoid organs [11] and more recently, by natural killer cells [12], where TSH-TSHR signaling is thought to mediate processes including T cell maturation, and phagocytosis and cytokine production in dendritic cells.

We have previously demonstrated a reduction in circulating fetal thyroid hormones (T3, T4) in PRRSV infected fetal pigs, with serum T3 and T4 concentration inversely correlated with fetal viral loads [13]. A more substantial link between dysregulation of fetal hypothalamic-pituitary-thyroid (HPT) axis and the DRGA SNP was reported in a study following up on the GWAS, which identified a significant difference in circulating T4 between homozygous genotypes [14]. These results establish the direct relevance of this genomic region to thyroid function and metabolism.

The dysregulation of HPT axis suggests a fetal manifestation similar to non-thyroidal illness syndrome (NTIS), which in humans is classically defined by low circulating T3 and/or T4, and inappropriately low to normal thyroid-stimulating hormone (TSH) levels in response to pathogenic infection, or chronic illnesses [15]. Such suppression of the HPT axis can be driven by inflammatory cytokines [16] or activation of the hypothalamic-pituitary-adrenal (HPA) axis in response to stress [17, 18]. In NTIS, inflammatory cytokines, cortisol, and thyroid hormone levels are interconnected, with cytokine elevation associated with suppressed thyroid function and HPA axis activation [19–21]. In addition, we have previously identified upregulation of the fetal cytokine response following PRRSV infection [22], suggesting fetal cytokine upregulation as a potential driver of the NTIS-like fetal response to PRRSV infection.

Given the complicated interactions among cytokines, HPA axis, and HPT axis during fetal PRRSV-2 infection as a possible mechanism underlying differential fetal viability, *DIO2* and *TSHR* are good candidates for causal variants associated with the beneficial effects of the DRGA SNP. For example, the combination of *DIO2* and *TSHR* mutations could significantly affect TSH-stimulated DIO2 activity [23], potentially contributing to the DRGA SNP’s impact on fetal viability. To investigate the role of *DIO2* and *TSHR* further, we used animal samples and phenotypic data collected from two independent PRRSV-2 challenge trials involving pregnant gilts in late gestation, with trial-1 conducted in 2012 [24] and analyzed by Yang et al. GWAS [6], and trial-2 conducted in 2019 [14]. An overarching objective of the present study was to investigate genetic variants of *DIO2* in relation to fetal resilience, and cortisol and thyroid hormone dysregulation following PRRSV-2 infection in pregnant gilts. The specific objectives of the present study were to: (1) Characterize *DIO2* gene polymorphisms in the parental population (trial-2), expanding the search for potential variants near the DRGA SNP; (2) genotype an identified *DIO2* missense mutation in the fetal population from trial-1 and trial-2; (3) compare fetal genotypes of the *DIO2* missense mutation to determine whether fetal *DIO2* expression and PRRS disease outcomes differed by genotype by combining phenotypic data from trial-1 and trial-2; (4) investigate if any *TSHR* gene polymorphisms are in linkage disequilibrium (LD) with the *DIO2* missense mutation in the parents from trial-2; and (5) investigate a potential relationship among cortisol and thyroid hormone levels, and *DIO2* polymorphisms.

## Results

### Identification of SNPs in exons of *DIO2* and *in silico* assessment of identified SNPs

**Fig. 1:**
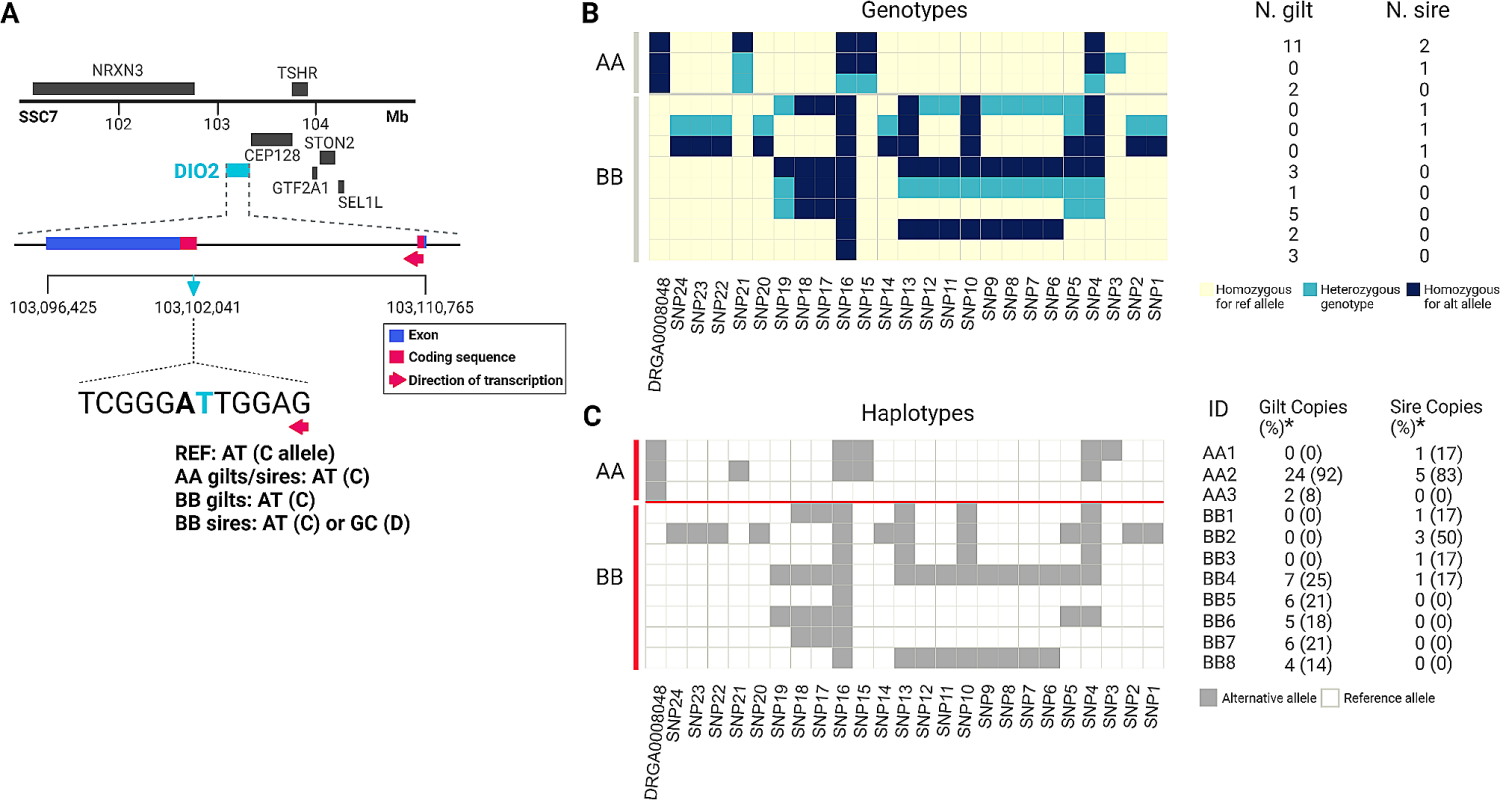
Identification of a missense variant (Asn91Ser) in porcine *DIO2*. (**A**) Sequenced exons are indicated in the context of DIO2 gene structure with neighboring genes on Sus scrofa chromosome (SSC7) 101–105 Mb region (Ensembl Release 105 (Dec 2021) used). Coding sequence (red colored box) encompasses the two sequenced exons (blue box). A single nucleotide variant site (light blue arrow) in the coding region on SSC7:103,102,041 is a missense variant changing asparagine (Asn) to serine (Ser) at position 91 of porcine DIO2 protein. This variant is present as a dinucleotide polymorphism with a synonymous variant (bolded left to the missense variant) on SSC7:103,102,040. AA or BB is genotype of DRGA0008048 (DRGA) SNP which is an intergenic variant ~ 14 Kb distal to *DIO2*. The alternative allele (GC) is labelled as D while the reference (REF) allele (AT) is C to distinguish the variant from DRGA. (**B**) Map of AA and BB genotypes for each SNP within the exonic region of *DIO2* in gilts and sires used in trial-2. SNP labels correspond to labels in additional file 1; SNP24-SNP3, SNPs in the 3 prime untranslated region; SNP1 and SNP2, Asn91Ser variant and the neighboring synonymous variant. Each cell denotes genotype at each SNP site. Light yellow cells, homozygous genotype for reference (ref) allele. Sky blue cells, heterozygous genotype. Dark blue cells, homozygous genotype for alternative (alt) allele. Number of gilts and sires was shown for each row indicating a combination of all genotypes across SNP loci. (**C**) Haplotypes in the sequenced *DIO2* region in AA versus BB genotype by DRGA SNP. The most probable haplotypes were determined by manual assignment. * denotes frequency of each copy of haplotypes within DRGA genotype (AA versus BB) and within parents (gilts versus sires). Gray cells, alternative allele. White cells, reference allele. Edited with BioRender

To characterize SNPs in *DIO2*, we performed Sanger sequencing using parental DNA samples (N = 33, 27 gilts, 6 sires) from trial-2. These parents were differentiated based on their specific DRGA genotypes (AA and BB) (Fig. 1A). We focused on the two exons encompassing the coding region (Fig. 1A), segments of *DIO2* that encode DIO2 protein. A total of 24 SNPs were detected across the sequenced exons, where 2 SNPs (rs333347361, p.Asn91Ser; rs323844025, p.Asn91=) were identified in the coding region from 2 BB sires (Fig. 1A), and the rest of the SNPs were located in the 3’ untranslated region (UTR) (Supplementary Table 1; additional file 1). As indicated, the dinucleotide polymorphism changes Asparagine (Asn) to Serine (Ser) at position 91 of the DIO2 protein sequence (ENSSSCP00000046993.1). One sire was heterozygous for this polymorphism (AT/GC) and the other was homozygous for the non-reference allele (GC/GC) giving the genotype notation, CD and DD, respectively (Fig. 1A).

Sequence analyses were conducted to assign SNP genotypes and derive haplotypes for the parental populations in the study (Fig. 1B and C). Haplotypes are blocks of linked SNPs that tend to be inherited together. Using the correlated patterns of the *DIO2* genotypes, we manually assigned haplotypes. The results showed that the *DIO2* sequences differ between the DRGA genotypes, AA and BB. Only 3 haplotypes were identified in AA parents, where haplotype 2 (AA2) was the most common. None of these haplotypes were found in the BB parents, where 8 haplotypes were identified with one of these (BB2) being defined by the dinucleotide polymorphism (allele D) (Fig. 1C). Five SNPs within the 3’ UTR (SNP14, 20, 22, 23, 24) were in complete LD with the two coding variants.

Next, we annotated all the SNPs in *DIO2* using pCADD scores to assess the functional impact of each (Supplementary Table 1; additional file 1) and compared the scores with those of variants proposed to be causally associated with carcass, developmental and disease traits (Supplementary Table 2; additional file 1). Variants causally associated with carcass or developmental traits were highly ranked (pCADD > 10), whereas variants causally related to pig response to external stimulus such as virus (porcine circovirus type 2) scored low (~ 0). The pCADD scores for the Asn91Ser mutation (p.Asn91Ser) and the neighboring synonymous variant (p.Asn91=) were low, 0.001 and 0.442 respectively, compared to most of the putative causative mutations. The highest pCADD scores amongst the 24 SNPs identified in the *DIO2* region were 10.244 (SNP5) and 9.568 (SNP22) (Supplementary Table 1; additional file 1). These SNPs are located in the 3’ UTR of *DIO2* and the SNP with the second highest pCADD score (SNP22) was in complete LD with the two coding variants (Fig. 1C). A further five 3’ UTR SNPs (SNP14, 20, 22, 23, 24) in complete LD with the Asn91Ser variant were part of the haplotype BB2 found in two of the three BB sires used in trial-2. Note that SNP5, along with SNP10 and SNP13 were also included in this haplotype, although they were not in complete LD with the coding variants. Despite its low pCADD score, the Asn91Ser mutation was chosen for further investigation for two key reasons. First, it represents the distinct haplotype (BB2), making its functional impact easier to interpret based on the predicted protein sequence change. Second, this variant selection approach was more feasible than sequencing the entire *DIO2* and *TSHR* regions in fetal pigs with potentially degraded DNA. The autolyzed fetuses, excluded from the trial-1 GWAS which identified DRGA SNP due to low DNA yield and quality [6], necessitated a method like TaqMan genotyping, which requires shorter DNA fragments compared to Sanger sequencing. We hypothesized that this haplotype, captured by the Asn91Ser mutation, may explain variation in fetal response in PRRSV infected gilts at late gestation. Additionally, no 3’ UTR variants were detected at the in-frame UGA codon or within the predicted Sec insertion sequence (SECIS) element. These elements are crucial for the incorporation of Sec into the DIO2 protein, ensuring its proper function [7].

### Association tests of genotype at the Asn91Ser mutation site with fetal *DIO2* expression level

Once all fetal genotypes for the Asn91Ser mutation were determined from a subset of trial-2 fetuses, we sought to identify whether genotype of Asn91Ser variant or other variants in LD with the Asn91Ser mutation affects *DIO2* gene expression level in fetal heart (HRT) and kidney (KID) from trial-2. Previous research examining the impact of PRRSV infection on the suppression of cell division by CDKN1A identified the HRT and KID as the most severely impacted tissues [25]. Thus, *DIO2* expression levels in these two organs were regressed on genotype.

**Fig. 2:**
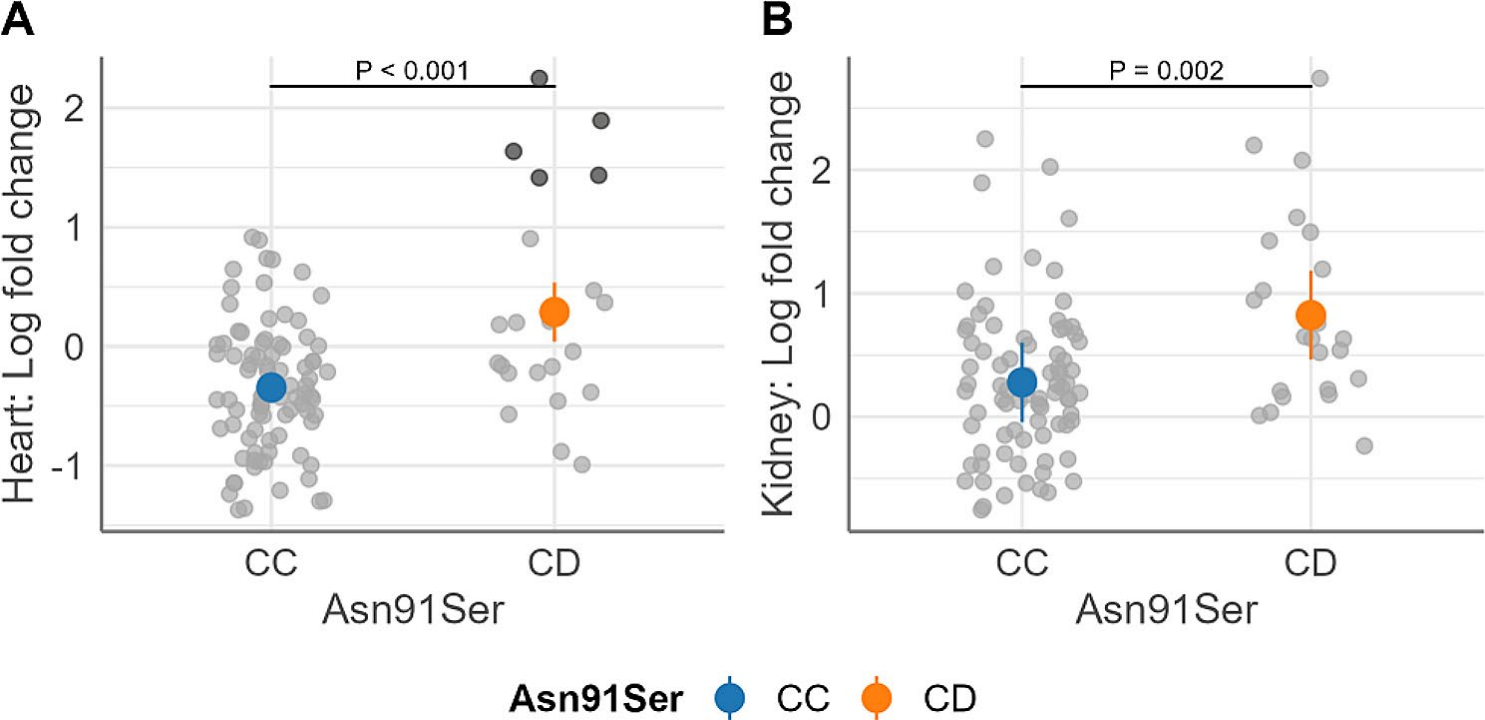
Estimated marginal means (EMM) of natural log transformed fold change (FC) of fetal *DIO2* expression. (**A**) fetal heart; (**B**) fetal kidney. Data is grouped by Asn91Ser genotype for the subset of the trial-2 [14]. Fold change was calculated relative to control fetuses from non-inoculated litters and transformed by natural logarithm. Colored dots and associated vertical lines are EMM and 95% confidence interval, respectively. Overlapping gray dots are observed values. 5 CD fetuses deviating over log FC > 1 are highlighted with the dark gray dots in (A). P values for coefficient of genotype are labeled in the plots. Fetal counts in each genotype: (A) CC = 87, CD = 23; (B) CC = 81, CD = 23. Ratio of brain to liver weight (*P* = 0.001) or brain weight (g) (*P* = 0.015) included as a covariate in the regression model for heart log FC or kidney log FC, respectively

There was a significant genotypic effect on *DIO2* expression in fetal HRT and KID (Fig. 2A and B), with higher expression in CD fetuses compared to CC fetuses. The estimated marginal mean (SE) on natural log transformed fold change (FC) by genotype were − 0.34 (0.06) for CC and 0.29 (0.12) for CD in fetal HRT (*P* < 0.001), and 0.28 (0.11) for CC and 0.82 (0.17) for CD in fetal KID (*P* = 0.002). Five CD genotyped fetuses with natural log transformed FC greater than 1 (Fig. 2A; darker gray dots) were identified as driving a large proportion of the variant effect in HRT based on finding that exclusion of these fetuses from the regression model reduced the coefficient by 59%. To get a sense of the underlying trend in these five CD fetuses versus the others, we compared their phenotypes. There were no numerically remarkable differences in phenotypic features specific to these five CD fetuses compared to other fetuses within the same range of the covariate (brain to liver ratio, 1.1 to 1.46), except they showed numerically higher viral loads and lower T4 level than other groups (Supplementary Table 3; additional file 1). This result suggests that the Asn91Ser mutation or its haplotype may alter the response phenotype, such that changes in *DIO2* expression are most evident in severely infected fetuses.

### Association tests of genotype at the Asn91Ser mutation site with fetal phenotypes

Because the genotype partly explained variation in *DIO2* mRNA expression in fetal HRT and KID from a subgroup of trial-2 fetuses, we further hypothesized that the Asn91Ser variant might be associated with changes in fetal viability, that were associated with DRGA SNP in trial-1 GWAS. We therefore sought to identify whether genotype of Asn91Ser substitution or other variants in LD with the Asn91Ser mutation affected fetal viability, survival, viral loads, thyroid hormone levels, and fetal growth using the combined subset of trial-1 and trial-2 fetuses (Supplementary Table 4; additional file 1).

#### Effect of genotype on fetal viability and survival

**Fig. 3:**
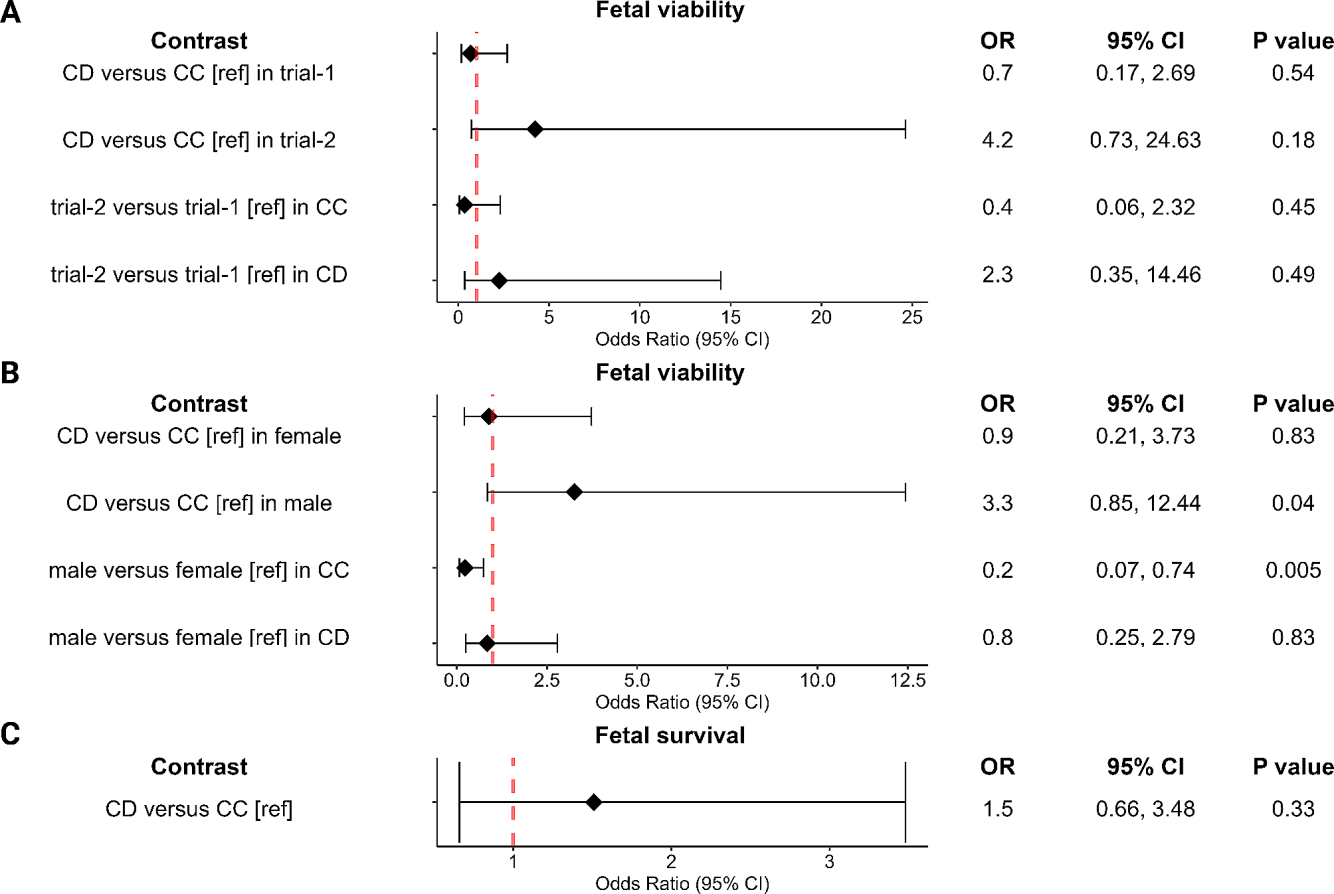
Estimated odds ratios (OR) and 95% confidence interval (CI) of fetuses being viable or live. CC is homozygous genotype for the reference allele for Asn91Ser mutation of *DIO2*, CD for heterozygous genotype. Red dotted vertical lines indicate OR = 1. The Benjamini-Hochberg (BH) adjusted P values are applied to all possible pairwise comparisons (*N* = 6) of levels of Asn91Ser and trial (**A**), and Asn91Ser and fetal sex (**B**). CC genotype or trial-1 was used as the reference group (ref). Different x-axis scales were used across figures. (**A**) Contrasts for the interaction between Asn91Ser (CD vs. CC) and trial (trial-2 vs. trial-1) for fetal viability. Trial-1, Yang et al’s GWAS [6]; trial-2, follow-up study to Yang et al.’s GWAS [14]. Fetal counts in each genotype within each trial: CC = 41, CD = 37, in trial-1; CC = 71, CD = 74, in trial-2. (**B**) Contrasts for the interaction between Asn91Ser (CD vs. CC) and sex (male vs. female) for fetal viability. Fetal counts in each genotype within each sex: CC = 57, CD = 47, in female; CC = 55, CD = 64, in male. (**C**) Contrast for Asn91Ser (CD vs. CC) for fetal survival

The genotype was not a significant predictor for fetal viability or survival, however, there were significant interactive effects estimated between genotype and trial (*P* = 0.03), and genotype and fetal sex (*P* = 0.04) for fetal viability (Fig. 3A and B). Post hoc analyses of the interacting factors revealed that the estimated probability (95% CI) of fetuses being viable was higher in CD fetuses versus CC fetuses (64.4, 95% CI 45.1–79.9% vs. 35.7, 95% CI 20-55.2%; adjusted *P* = 0.04) only within males, and male fetuses had lower probability of being viable than female fetuses only in the CC genotype group (35.7, 95% CI 20-55.2% vs. 70.6, 95% CI 51.6–84.4%; adjusted *P* = 0.005). There were no significant interactions between genotype and trial, and genotype and sex for fetal survival (Fig. 3C).

#### Effect of genotype on viral loads and thyroid hormone levels

**Fig. 4:**
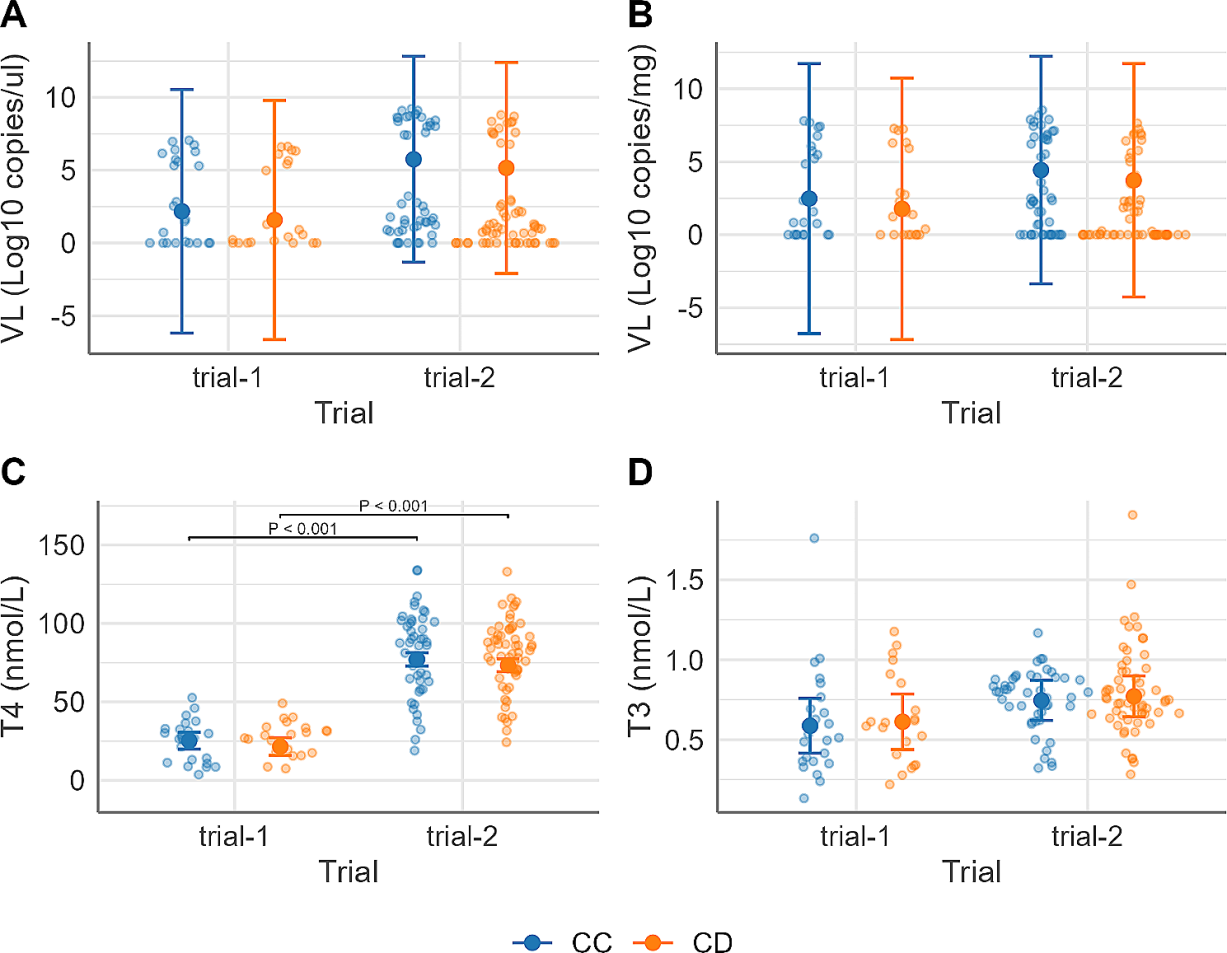
Estimated marginal means (EMM) of fetal viral loads and circulating thyroid hormones by Asn91Ser genotype (CC, CD) for each trial (trial-1 [6], trial-2 [14]). (**A**) viral load (VL) in serum (log10 copies/µl); (**B**) viral load (VL) in thymus (log10 copies/mg); (**C**) T4 level (nmol/L); (**D**) T3 level (nmol/L). Large central dots and associated vertical lines represent the EMM and 95% confidence interval, respectively. Overlapping small dots are raw data. Different y-axis scales used between (**C**) and (**D**). P values for coefficients of genotype or trial are shown in the plots if significant at *P* < 0.05. Fetal counts in each genotype within each trial: CC = 24, CD = 21, in trial-1; CC = 47, CD = 59, in trial-2 (1 CD fetus from trial-2 excluded for T3 level after residual analyses)

The genotype was not associated with viral load in serum and thymus (Fig. 4A and B) nor circulating thyroid hormone levels (T3 or T) (Fig. 4C and D). No significant interactions between genotype and other categorical predictors were identified for these traits.

#### Effect of genotype on fetal growth

**Fig. 5:**
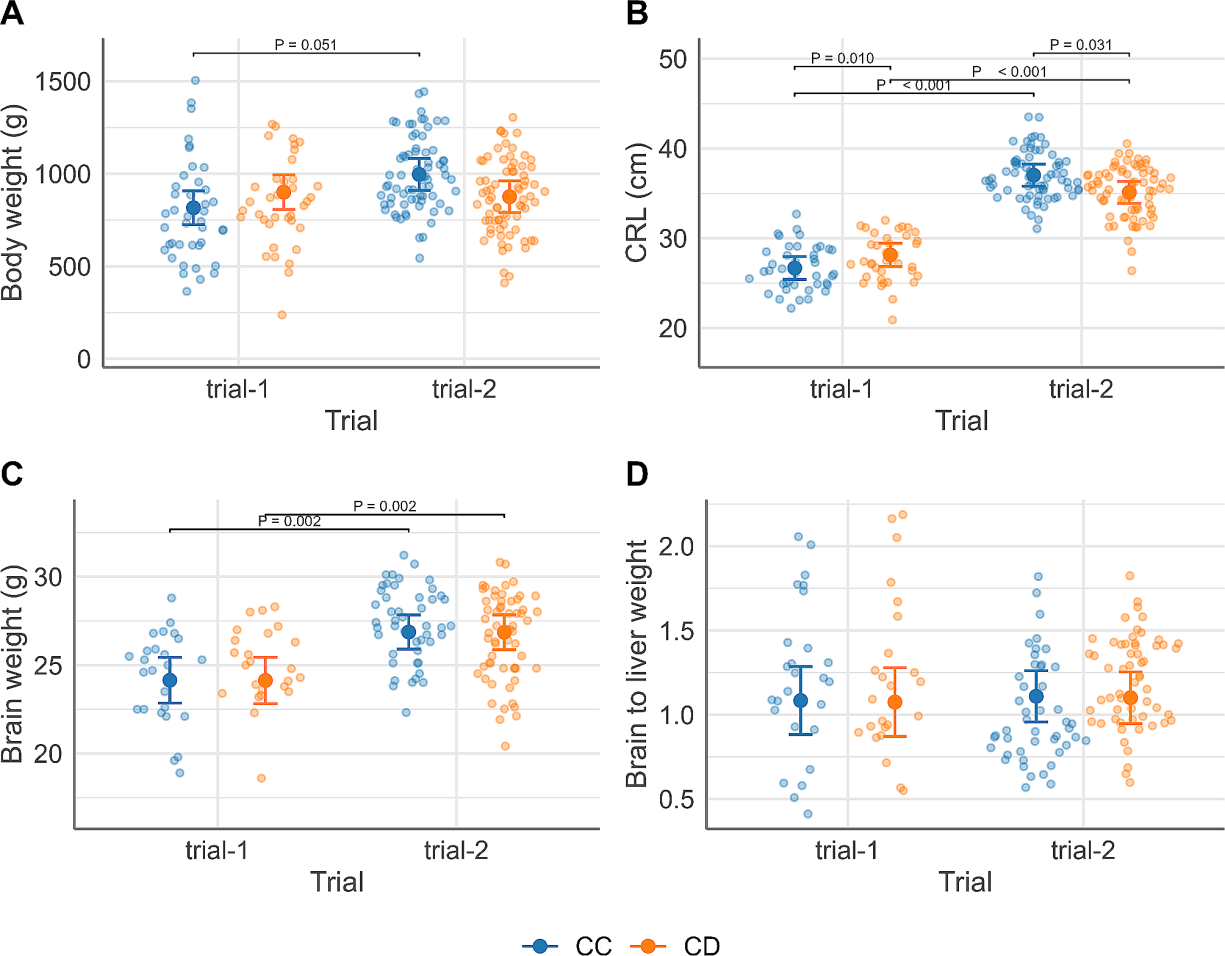
Estimated marginal means (EMM) of fetal growth by Asn91Ser genotype (CC, CD) for each trial (trial-1 [6], trial-2 [14]). Large central dots and associated vertical lines represent the EMM and 95% confidence interval, respectively. Overlapping small dots are raw data. (**A**) fetal body weight (g); (**B**) fetal crown rump length (CRL) (cm); (**C**) fetal brain weight (g); (**D**) fetal ratio of brain to liver weight. (**A, B**) The Benjamini-Hochberg (BH) adjusted P values are applied to all possible pairwise comparisons (*N* = 6) of levels of Asn91Ser and trial and labeled for the genotypic or trial effects at *P* < 0.10) (**C, D**) P values for coefficients of genotype or trial are shown in the plots if significant at *P* < 0.05. Fetal counts in each genotype within each trial: (A, B) CC = 41, CD = 37, in trial-1; CC = 71, CD = 74, in trial-2; (C, D) CC = 26, CD = 24, in trial-1; CC = 47, CD = 59, in trial-2 (1 CD fetus from trial-2 excluded for the ratio of brain to liver weight after residual analyses)

Fetal growth measured by body weight, crown rump length (CRL), brain weight and the ratio of brain to liver weight was not significantly different between CC and CD genotype, but an interaction effect existed between genotype and trial for fetal body weight (*P* = 0.007) and CRL (*P* = 0.002), respectively, (Fig. 5A and B). CC fetuses tended to be heavier in trial-2 versus trial-1 (996.4 ± 39.1(SE) g vs. 816.2 ± 43.6 g, adjusted *P* = 0.051), but this tendency was not present for CD fetuses. CD fetuses had longer CRL than CC fetuses in trial-1 (28.2 ± 0.6 cm vs. 26.7 ± 0.6 cm, adjusted *P* = 0.010) whereas CD fetuses had shorter CRL than CC fetuses in trial-2 (35.1 ± 0.6 cm vs. 37.0 ± 0.6 cm, adjusted *P* = 0.031). For brain weight and the ratio of brain to liver weight, no significant interactions were present between genotype and trial (Fig. 5C and D). Additionally, there were no significant interaction effects between genotype and fetal classification for all the fetal growth measures.

### Identification of variants in exons of *TSHR* in sires

Since the Asn91Ser mutation was only present in two BB sires in trial-2, Sanger sequencing of the *TSHR* coding region of trial-2 sires was conducted to find any *TSHR* variants in LD with the Asn91Ser variant. The receptor encoded by this gene is a critical component of the HPT axis and is also located ~ 68.7 kb for the DRGA SNP. Thirty-three variants were detected in the sequenced region: 11 5’ UTR variants (10 SNPs, 5 bp-insertion); 18 intronic variants (16 SNPs, 2 bp-insertion, 12 bp-insertion); 3 coding variants (2 synonymous SNPs, 1 missense SNP); and 1 3’ UTR variant (Supplementary Table 5; additional file 1). Twenty-one variants were estimated to be in moderate to high LD with the Asn91Ser mutation (estimated by R² (allelic correlation coefficient) > 0.4). All sires had the same genotype for the missense SNP, thus, they were not differentiated by Asn91Ser variant. However, sires were differentiated by genotype at the 2 synonymous SNP sites (R² = 0.47). All BB sires had at least one variant allele at these loci, with 2 BB sires (CD, DD sires) homozygous for the variant alleles. Interestingly, one of the three intronic variants in highest LD with the Asn91Ser variant (R² = 0.67) was a 12-bp insertion specific to the 2 BB sires carrying the D allele. Overall, these findings demonstrated that there were mutations in *TSHR* exons in LD with Asn91Ser variant, specific for 2 BB sires carrying the D allele, suggesting the *DIO2*-*TSHR* region was genetically differentiated between AA and BB parents in trial-2.

### Serum cortisol levels by genotype and fetal classification

**Fig. 6:**
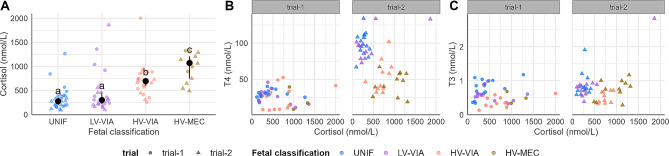
Fetal cortisol, T4, and T3 levels in sera by fetal classification and trial. (**A**) Data from both trials (trial-1 [6] and trial-2 [14]) were merged into each fetal classification. Asn91Ser genotypes were also combined at each level of fetal classification due to lack of significant differences on cortisol levels stratified by trial or fetal classification. Large central dots and associated vertical lines represent median values and equi-tailed two-sided nonparametric 95% confidence intervals, respectively, for each fetal classification. Overlapping small dots are raw data. Different superscripts denote a significant difference in median values between groups, based on Benjamini-Hochberg (BH) adjusted P value < 0.05 from pairwise two-sample permutation tests across groups of fetal classification. (**B, C**) Fetal observations for cortisol and T4 or T3 level colored by each group of fetal classification with different shapes for trial, respectively. Fetal counts in each level of fetal classification across trials: UNIF = 28, LV-VIA = 25, HV-VIA = 23, HV-MEC = 14

To further understand the variant effect on *DIO2* expression level in fetal HRT and KID, cortisol concentration in fetal sera was assessed for a subset of fetuses used for association tests. We hypothesized that the observed differences in *DIO2* expression between Asn91Ser genotypes may be related to a differential stress response in fetal HRT or KID according to the genotype. We used the non-parametric permutation test due to the small sample size per genotype within fetal classification (3–9 fetuses per each genotype within fetal classification). There was no significant difference in median values of fetal cortisol levels between genotype when controlled for trial (*P* = 0.76). We also found no significant difference in the fetal cortisol levels between CC and CD across different fetal classification (*P* = 0.75). However, in conducting this analysis we demonstrated that median cortisol level was significantly higher in highly PRRSV-infected fetuses (HV-VIA or HV-MEC) compared to viable fetuses that were uninfected (UNIF) or had low viral load (LV-VIA), independent of genotype, while accounting for variability across different trials (Fig. 6A). This finding provides evidence that fetal PRRSV-2 infection induces a stress response, and this response is related to infection severity as indicated by fetal classification. Additionally, fetal cortisol, T4 and T3 levels in sera showed clustering patterns by fetal classification, specifically in HV-VIA and HV-MEC versus UNIF or LV-VIA (Fig. 6B and C).

## Discussion

To the best of our knowledge, this is the first examination of a missense variant (p.Asn91Ser) and its relevant haplotype in the porcine *DIO2* gene relative to fetal responses to PRRSV or other physiological responses. The missense mutation (Asn91Ser) in *DIO2* was in LD with the DRGA SNP that was identified to be associated with fetal viability in the trial-1 GWAS [6] but only presented in ~ 4% of the fetal population (37 heterozygous CD fetuses in a total of 926 fetuses used in the GWAS). Relative *DIO2* mRNA levels in fetal heart and kidney were significantly related to the Asn91Ser variant with the heterozygous genotype having increased levels of *DIO2* expression in the heart and kidney in comparison to the homozygous genotype. Other fetal outcomes, which include fetal viability, survival, viral load in serum or thymus, circulating cortisol and thyroid hormone levels (total T4 and T3), and fetal growth measures, were not found to be significantly associated with the Asn91Ser mutation.

Our results indicate the Asn91Ser variant, or its haplotype, does not have a strong influence on fetal phenotypic response to PRRSV-2 infection although there were significant interactions between genotype and sex for fetal viability, and between genotype and trial for body weight and crown rump length (CRL). Male CD fetuses had a higher estimated probability of being viable compared to male CC fetuses. Conversely, there were no significant differences in viability between CD and CC genotypes in female fetuses. Additionally, male fetuses in the CC genotype group had a lower probability of viability compared to female fetuses in the same group. It has been hypothesized that there is differing sensitivity to environmental stress between sexes especially with regards to prenatal growth and development, leading to sexually dimorphic patterns [26]. For instance, maternal malnutrition can induce intrauterine fetal growth restriction that is fetal sex dependent, with female fetuses more protected than male fetuses [27, 28]. Therefore, this might partially explain lower viability rate observed in male versus female fetuses. Thus, our findings suggest that the Asn91Ser mutation might modulate sex dimorphism or fetal sex may differentially confer the variant effect in response to PRRSV-induced fetal stress. We also observed significant interaction effects between genotype and trial for CRL. CD fetuses had longer CRL than CC fetuses in trial-1 but shorter CRL in trial-2. These opposite genotype effects may reflect interactions among genetic and nongenetic factors, including maternal/paternal effects and trial/batch-specific variations.

Interestingly, based on DIO2 protein sequence alignment across multiple species (Additional file 2), the Asn91Ser mutation is found to be located just 1 residue upstream of a putative destruction loop, comprised of 18 amino acid residues (92–109 position) of porcine DIO2. Although this loop is shown to be less conserved compared to other regions of DIO2, such as an activity center highly conserved for the catalytic function of deiodinases, across multiple species (122–136 position of porcine DIO2), the destruction loop has been demonstrated to be the functional domain involved in the post-translational regulation of DIO2 activity mediated by the ubiquitin-proteasome system [29]. This destruction loop confers an intrinsic instability of DIO2 by binding with the WD (tryptophan – aspartic acid) repeat domain of the WD repeat and SOCS box-containing protein 1 (WSB1), which in turn mediates proteasomal degradation of DIO2 [29]. Notably, a single nucleotide substitution at position 92 of human DIO2 (Thr92Ala), i.e. the first residue of the destruction loop, has been demonstrated to alter enzymatic characteristics of DIO2, including longer half-life (~ 2.5 fold increase) [30], greater stability [31], less efficient T4 to T3 conversion [31] or decreased in vivo T4 5’ deiodination [32]. However, the Asn91Ser variant may not have any major impact on porcine DIO2 enzymatic characteristics and related cellular changes, as it was predicted to have no deleterious effect based on the pCADD score. Thus, it may suggest that the Asn91Ser variant itself is likely to be a conservative substitution with little functional significance in the context of fetal PRRS outcomes. Nonetheless, there is increasing evidence that conservative mutations, which induce substitutions between amino acids with similar chemical properties (as here), may contribute to differences in complex traits or may have an impact on protein function. For instance, the isoleucine (I) to valine (V) substitution at position 199 of the protein kinase AMP-activated non-catalytic subunit gamma 3 (PRKAG3) in pigs significantly affected meat quality traits [33]. In humans, a recent study indicated that an Asn to Ser substitution at position 232 of the protocadherin 19 (PCDH19) was predicted to abolish a hydrogen bond among the variant site and two other amino acids at position 320 and 323 of the PCDH19, suggesting molecular interactions could be changed by the substitution [34]. It has also been shown that mutant proteins which were altered from Asn to Ser or vice versa located at a highly conserved residue were dysfunctional compared to the wild type proteins such as an adenosine triphosphate (ATP) synthase subunit [35] and a glutamate transporter [36]. Thus, it would be interesting to test if the Asn91Ser mutation of DIO2 may exert an effect at the protein level, for instance, by interacting with the destruction loop or influencing local properties of the loop.

Sequence analyses of sires with the D allele showed a differentiated haplotype comprising of five SNPs within the 3’ UTR in complete LD with the two coding variants (p.Asn91Ser and p.Asn91=) of the *DIO2* gene, when compared with haplotypes of non-carriers (CC animals). Interestingly, pCADD scores across the five 3’ UTR variants in this haplotype were estimated to be higher (ranged from 9.56775 to 0.05295) than that of the missense variant. Among these 3’ UTR SNPs, SNP22 exhibited the second highest pCADD score. Additionally, SNP5, the highest pCADD scored SNP in our study, was in partial linkage with the Asn91Ser mutation. Thus, these predictions may potentially suggest a presence of a combined effect of this distinct haplotype on *DIO2* gene regulation between CC and CD fetuses, which may underlie the genotypic differences in heart and kidney *DIO2* mRNA levels observed in our study. 3′ UTR *cis*-elements in a given mRNA are known to affect gene expression by interacting with *trans*-acting factors such as RNA binding proteins (RBPs) or microRNAs (miRNAs), which in turn regulates the stability, subcellular localization, and translation of the mRNA [37, 38]. Therefore, sequence alterations at the five 3’ UTR SNP loci in complete LD with the Asn91Ser mutation, along with SNP5 locus, may create perturbations in interactions with RBPs or miRNAs, thereby influencing mRNA stability or degradation. This may explain the observed difference in *DIO2* mRNA level by the genotypes of the Asn91Ser substitution. Additionally, haplotype-specific secondary structure of the *DIO2* mRNA may exist, which may be involved in a difference in the mRNA stability or half-life thus affecting mRNA levels. For instance, one of the causal mutations located in the coding region of myosin heavy chain 7 (MYH7) gene linked to hypertrophic cardiomyopathy has been demonstrated to modify the secondary structure of the *MYH7* mRNA [39]. Thus, it suggests that single nucleotide changes in a gene may influence structural characteristics of a given mRNA which relates to alterations in the mRNA stability. Additionally, CD genotype related to higher *DIO2* expression may be explained by allelic differences in the 3’ UTR SNPs which may cause different usage of polyadenylation signal(s) in the 3’ UTR. This process may be involved in differences in mRNA length and stability. For instance, a single nucleotide change (rs80800372) in the 3’ UTR of the guanylate binding protein 1 (GBP1) gene linked to PRRSV viremia and weight gain was related to the variation in the 3’ UTR length due to different usage rate of the proximal polyadenylation signal in the 3’ UTR [40]. In this study, the A allele of the mutation was associated with higher *GBP1* expression by elevating polyadenylation from the proximal polyadenylation site, which produced higher proportion of the shorter, thus presumably more stable, *GBP1* mRNA. This was linked to the additive effect of A allele in *GBP1* mRNA levels.

There may also have been non-genomic mechanisms related to the observed genotypic differences. For instance, it may indicate that a differential compensatory response occurs in response to different levels of local T3 availability in fetal heat and kidney between CC and CD fetuses since T3 is capable of directly suppressing *DIO2* mRNA level as one of the pre-translational regulatory mechanisms, whereas T4 and rT3 downregulate DIO2 at the post-translational level [41], thus suggesting the observed effect may be independent of the genotype.

Two distinct features of the *DIO2* at the mRNA level are an in-frame UGA stop codon which can be recognized as selenocysteine (Sec) and a Sec insertion sequence (SECIS) element in the 3’ UTR forming a stem-loop RNA structure [7]. The SECIS element is essential for the UGA codon to be translated as Sec, where this SECIS element binds to *trans*-acting factors such as SECIS binding protein 2 (SECISBP2) which also interacts with an elongation factor (eEFsec) specific for selenocysteyl-tRNA (tRNA^Sec^) [7]. The complex interactions among the SECIS element, SECISBP2, eEFsec, and tRNA^Sec^, with other known factors ultimately result in the incorporation of Sec in the active site of the DIO2 enzyme [7], making DIO2 one of the selenoproteins in vertebrates. In our screening of genetic variants using Sanger sequencing in dams and sires, no variants were detected at the UGA codon nor within the predicted type II SECIS element with 75 bp length. Thus, it is likely that there was no possible molecular disruption in the process of the Sec insertion into fetal DIO2 protein directly attributable to the mutations found in the *DIO2* locus.

Finally, it should be noted that our study could not provide a broader view for the effect of the Asn91Ser variant on fetal disease response using three possible genotypes (CC, CD, DD) since all the included fetuses inherited the variant allele only from their sires (CD or DD), with their dams having no variant allele (CC). Thus, testing for the association between Asn91Ser variant or other variants in LD and fetal phenotypes following maternal PRRSV-2 infection could not be performed under a full genetic model such as recessive (CC + CD versus DD), additive (CC versus CD versus DD), or dominant (CC versus CD + DD) model [42]. Furthermore, the animal random genetic effect to adjust for potential genetic relatedness other than the Asn91Ser locus among tested fetuses could not be included in the regression models due to lack of additional genomic data, which may make the estimated allelic effect less confident and potentially biased.

Separate from the identification of *DIO2* mutation effects, our study provides evidence that maternal PRRSV-2 infection in late gestation can cause disruption in hypothalamic-pituitary-adrenal (HPA) axis and the hypothalamic-pituitary-thyroid (HPT) axis in highly infected fetuses, based on the observations of increased cortisol level and decreased thyroid hormone levels in sera of fetuses with high virus level in their thymus. This indicates that fetal response to PRRSV is related to the dysregulation of both HPA and HPT axis. Our previous finding indicates that meconium staining of skin is a distinct indicator of compromise in response to fetal PRRSV-2 infection [24]. The present study shows that increased severity of fetal PRRSV-2 infection is associated with increased cortisol level in fetal serum as observed between the HV-VIA and HV-MEC groups. Thus, these two findings suggest meconium-stained fetuses as being more physiologically stressed than UNIF, LV-VIA, or HV-VIA fetuses following PRRSV-2 infection.

Periparturient cortisol rise is considered a prerequisite to initiate parturition in livestock and allow for terminal maturation necessary for postnatal survival. It has been shown that total corticosteroid concentration in fetal pig sera starts to drastically rise within the 24 h before parturition [43]. Activation of the fetal HPA is a critical factor for induction of the parturition cascade [44]. For instance, premature delivery can be induced when fetal pigs were injected with a synthetic corticosteroid, dexamethasone, on gestation day 102, leading to a reduction in the length of gestation [45]. Cortisol-dependent maturational changes in fetal organs are also necessary before farrowing [46]. The rise in fetal pig cortisol towards birth is accompanied by rapid morphological growth in fetal adrenal cortex during the last week of gestation, where fetal adrenal weight increases largely due to hyperplasia in the zona fasciculata which secretes cortisol [47]. Furthermore, increasing cortisol level in fetal pigs towards birth results in accumulation of glycogen in fetal liver and muscle [48, 49], providing energy sources after birth [49].

Thus, we hypothesize that the increase in fetal cortisol level due to PRRSV infection in late gestation may disrupt normal periparturient processes in fetal pigs, including those related to induction of farrowing. This may contribute to a mechanism inducing early farrowing, abortion, and even fetal death in pregnant female pigs infected by PRRSV in late gestation (after day 72). If there are many highly-infected (stressed) fetuses in the litter, the cumulative effects of increased cortisol production in the fetuses could result in premature farrowing (observed in the form of spontaneous abortion). However, if there are insufficient highly-infected (stressed) fetuses in the litter to induce farrowing, then the individual fetuses may die prematurely as a consequence of viral infection.

## Conclusion

Our results did not support the hypothesis of an effect of the Asn91Ser substitution or its haplotype on fetal phenotypic response in PRRSV infected gilts, but this variant was related to higher *DIO2* expression levels in fetal heart and kidney in CD versus CC fetuses, possibly driven by severe fetal infection of a few CD fetuses. Interestingly, among male fetuses, the CD genotype was more likely to be viable than the CC genotype. Thus, the present study suggests that a complex relationship among *DIO2* genotype, *DIO2* expression, fetal sex, and fetal viability may exist during the course of fetal PRRSV infection. Despite the absence of a direct association between the Asn91Ser mutation and alterations in circulating cortisol, T4, and T3 levels, elevated cortisol levels were observed in severely affected fetuses following PRRSV infection. Thus, it suggests the increase in cortisol levels, indicative of fetal stress response, may lead to fetal complications, such as fetal compromise, fetal death, or premature farrowing, during PRRSV infection.

## Methods

### PRRSV challenge experiments and collection of fetal phenotypes

The two pregnant gilt PRRSV-2 challenge trials (trial-1 and trial-2) were conducted as previously described [24]. Both trials were approved by the University of Saskatchewan Animal Research Ethics Board and conducted in adherence with the Canadian Council on Animal Care guidelines for humane animal use (AUP #20110102 for trial-1; #20180071 for trial-2). Animals were purchased from Fast Genetics Inc. (Spiritwood, Canada) for both trials. For trial-1 [24] and trial-2 [14], purebred Landrace (LR) gilts and Yorkshire (YORK) sires were used to produce litters. The DRGA genotype frequencies in trial-1 were ~ 46% AA, ~ 42% AB and ~ 12% BB in LR dams (total 114 virus infected gilts) and ~ 79% AA, ~ 21% AB and 0% BB in the YORK sires (total 24 sires), resulting in a diverse fetal population for the DRGA SNP. For trial-2, 27 LR gilts and 6 YORK sires were selected for planned breeding based on their DRGA SNP genotypes (AA × AA or BB × BB) to produce homozygous DRGA litters of purely AA and BB fetuses in 6 batches (N = 4–5/batch). LR gilts were inoculated with 1 × 10^5^ TCID_5_ PRRSV-2 (NVSL 97-7895) on gestation day 85 ± 1 for trial-1 (N = 114) and 86 ± 0.9 for trial-2 (N = 22). Control gilts were similarly mock-inoculated with minimum essential media (N = 19 in trial-1, N = 5 in trial-2). In both trials, gilts and fetuses were euthanized 21 days post inoculation (dpi) by intravenous barbiturate (Euthanyl Forte, Bimeda-MTC, 77 mg/kg pentobarbital sodium), followed by assessment of fetal phenotypes, dissection of fetuses and sample collection during necropsy (fetal phenotypes are indicated in the phenotypic association analyses section below).

### Study animals, DNA extraction, PCR and Sanger sequencing

The parental animals from trial-2 were used for DNA extraction and sequencing of *DIO2* exons to screen for genetic variation in the population. Genomic DNA was extracted from adrenal glands of gilts (13 AA, 14 BB genotypes) and ear tissue from sires (3 AA, 3 BB genotypes), using a commercial kit and following the manufacturer’s instructions (DNeasy Blood & Tissue Kit; Qiagen, Canada). To detect SNPs in the exons of *DIO2*, a total of 8 primer sets were designed to amplify the two exons harboring the coding sequence of *DIO2* (Supplementary Table 6; Additional file 1). PCR was conducted using Platinum II Hot-Start PCR Master Mix (2X) (Invitrogen, Canada) in a Veriti™ 96-Well Thermal Cycler (Applied Biosystems, Canada). Reaction composition for PCR was as follows: 10 ul of master mix, 1 ul of forward primer (10 uM), 1 ul of reverse primer (10 uM), 4 ul of GC enhancer, 4 ul of DNA (10ng/ul). Amplicons were purified using ExoSAP-IT™ PCR Product Cleanup Reagent (Applied Biosystems, Canada). Purified amplicons were separated and visualized on a 1% agarose gel to check amplification specificity, then cycle sequencing and capillary electrophoresis were conducted using purified PCR products sent to Macrogen Inc. (South Korea). Processing of raw sequencing chromatograms and variant analysis were completed using SeqMan (DNASTAR, Inc., version 7.0.0). Additionally, Sanger sequencing of the *TSHR* gene was conducted using the same methods to screen sequence variants in sires from trial-2 (N = 6, 3 AA, 3 BB) using primers described in Supplementary Table 6 (Additional file 1). All 11 exons including some portions of 5’ and 3’ untranslated region (UTR) were sequenced to cover the entire coding sequence of *TSHR*.

### Analyses of SNPs identified in DIO2

#### *In silico* prediction of the effect of identified SNPs

To narrow down candidate mutations impacting *DIO2* from the initial Sanger sequencing, we used combined annotation dependent depletion for pig (pCADD) [50] to predict the potential impact of identified SNPs on porcine DIO2 protein. This score was developed by Groß and colleagues [50] to aid with prioritization of single nucleotide variants (SNVs) by providing the probable deleteriousness of SNVs derived from a logistic regression model incorporating various genomic annotation resources. SNVs with score > 20 are distributed in the top 1% highest scored SNVs, and SNVs with score > 30 in the top 0.1% highest scored SNVs, with higher scores being more deleterious, ranging from ~ 95 to 0. pCADD scores for SNPs detected from Sanger sequencing were extracted from the publicly available link [50]. pCADD scores for known or proposed causal variants (referenced in [50, 51]) were first investigated to better understand the expected range of pCADD scores for well characterized porcine causal mutations (Supplementary Table 2; additional file 1). Additionally, a Sec insertion sequence (SECIS) element in the 3’ UTR of *DIO2* was predicted using the Selenoprotein prediction web server [52], and 3’ UTR SNPs identified were located relative to the predicted SECIS element, where all sequences obtained from Sanger sequencing were aligned with the predicted SECIS sequence.

#### Haplotype determination and linkage disequilibrium (LD) estimation

The most probable haplotypes were determined manually based on the observed genotypes of AA and BB parents in trial-2 to compare the architecture of genetic variation in the *DIO2* region between DRGA genotypes. Pairwise LD was estimated using squared allelic correlation coefficient (R^2^) between SNPs, using genetics (v1.3.8.1.3) in R (v4.1.0) [53].

### Fetal Asn91Ser genotyping

A dinucleotide polymorphism encoding a missense variant substituting Asparagine (Asn) for Serine (Ser) at position 91 of the DIO2 protein was selected to genotype fetuses using fetal spleen or thymus DNA samples archived from trial-1 and trial-2. To avoid confusion with the related DRGA genotypes (AA and BB), CC was used to denote the homozygous reference genotype (AT/AT), CD for the heterozygous genotype (AT/GC), and DD for the homozygous alternative genotype (GC/GC).

Custom TaqMan™ SNP Genotyping Assays (Applied Biosystems) and TaqMan™ Universal PCR Master Mix (Applied Biosystems) were used for genotyping reactions. Designed primers and probes were as follows: forward primer, 5’-AAGATGCACCCAATTCCAGTGT-3’; reverse primer, 5’-GTGGCACTCAGCTCCATCAA-3’; Taqman MGB probe (VIC labeled), 5’-TGCATGTCTCCAATC-3’; Taqman MGB probe (FAM labeled), 5’-CATGTCTCCAGCCC-3’. Reactions were run in duplicate with positive controls and a negative control in each plate using StepOnePlus™ Real-Time PCR System (Applied Biosystems, Canada). The allele discrimination plot was generated for each run and examined to assign fetal genotypes.

We first verified that the Asn91Ser mutation was historically present in the trial-1 fetal population [6]. Fetuses were selected to maximize the chance of capturing unique parental breeding (dam × sire) among the litters which produced AB or BB fetuses for the DRGA SNP since the D allele of Asn91Ser was only present in 2 BB sires, based on the Sanger sequencing results of trial-2 parents. A total of 69 fetuses were tested (56 AB, 13 BB) using an in-house TaqMan SNP genotyping assay. Only 3 fetuses (1 AB, 2 BB) heterozygous for the Asn91Ser mutation (CD genotype) were found. 3 fetuses were from the same AB sire, but from 3 different dams (1 unknown genotype, 1 AB genotype, 1 BB genotype). So, BB dam was separately sequenced, whose DNA was available at the time of experiment. This dam did not have D allele (CC genotype). Additionally, we confirmed that none of the fetuses from BB dams X AA sires (N = 16 fetuses/69 total tested fetuses) nor fetuses from AB dams X AA sires (N = 36/69) had the D allele. Therefore, these 3 CD fetuses were considered to have inherited the D allele from the single AB sire. Subsequently, entire litters from this sire (78 fetuses in total) were genotyped to enable an association analysis with fetal phenotypic traits. A subset of AA fetuses (N = 38) from AA × AA parents were also genotyped to verify the D allele was not present in AA population. We selected AA fetuses to maximize the chance of capturing unique parental breeding (AA dam × AA sire) among the litters which produced AA fetuses. We confirmed there was no D allele in the selected AA fetuses.

For trial-2 fetal pigs, since we only detected one DD sire bred to 5 BB dams in two batches, fetal genotypes from these batches were inferred from parental genotypes determined by Sanger sequencing. In these batches, all dams (N = 10, 5 AA, 5 BB) and two AA sires had CC genotypes, while a single BB sire had the DD genotype. Therefore, the breeding combinations in these batches were either CC dams X CC sires or CC dams X DD sire. Consequently, fetuses in these litters inherited only C alleles from dams, and either C or D alleles from their sires. All live fetuses (N = 59) were verified for their inferred CD genotype by TaqMan SNP genotyping assay.

### Gene expression analysis

To assess the effect of DRGA genotype and Asn91Ser mutation on fetal *DIO2* gene expression, RNA was extracted from fetal heart (HRT) and kidney (KID) samples from trial-2. A total of *n* = 110 heart and *n* = 104 kidney samples were selected from all live and infected fetuses in trial-2 PRRSV infected dams. Fetuses were selected if they had viral load > 0.7 log_10_ copies/µL or mg in serum and thymus and viral load > 0.4 log_10_ copies/mg in placenta, along with high RNA quality. Tissue was ground to a fine powder under liquid nitrogen and RNA isolated using Trizol and a double precipitation method as previously described [54]. Contaminating DNA was removed with the turbo DNA free kit (ABI) before RNA integrity was assessed by denaturing gel electrophoresis [55], with all samples displaying clean bands corresponding to 28s and 18s RNA at a ratio of 2:1. cDNA was generated from 2 ug of total RNA using the high capacity cDNA reverse transcription kit (Invitrogen) and gene expression measured using Sso advanced universal sybr green supermix (Biorad) and a CFX connect qPCR system (Biorad). Validated primer sequences specific for *DIO2* and 4 reference genes (*SDHA*, *STX5*, *ACTB*, *HMBS*) were taken from previous literature [13, 22] (Supplementary Table 7; additional file 1). Expression was normalized to the geometric mean of two stable housekeeping genes in each tissue and fold change calculated in relation to control fetuses from non-inoculated litters using the 2^−ΔΔCT^ method. For statistical analysis, fold changes were then natural log transformed to reduce the positively skewed observations for CD fetuses, and linear mixed-effects models were fitted to predict *DIO2* expression levels by the genotypes of the Asn91Ser substitution site by using the “lmer” function in lme4 (v1.1.27.1) [56] in R (v4.1.0) [53]. Covariates such as brain weight or ratio of brain to liver weight were included in the models since these were selected as predictors after backward eliminations of non-significant fixed effects (*P* > 0.05) of all possible explanatory variables using the “step” function in lmerTest (v3.1.3) in R (v4.1.0), while keeping random effects of experimental batch and gilt in the reduced models. Tested explanatory variables included fetal preservation, fetal sex, DRGA genotype, fetal weight, brain weight, and brain to liver ratio.

### Phenotypic association analyses

To determine if the genotype at the Asn91Ser mutation was associated with fetal responses following maternal PRRSV2 infection in late gestation, fetal phenotypes collected from the trial-1 and trial-2 experiments were regressed on the genotype.

Genotyped fetuses selected for association tests included 78 fetuses in trial-1 and 145 fetuses in trial-2 (Supplementary Table 8; additional file 1). The analysis included homozygous reference genotype (CC) and heterozygous genotype (CD) carrying the alternative allele for Asn91Ser mutation. No DD fetuses were included since there was only a single CD sire identified in trial-1 and the only DD sire in trial-2 was bred to CC gilts.

Fetal phenotypes for association tests included fetal viability and survival, viral load in serum and thymus, serum thyroid hormone levels (total T4, T3), and growth measurements (body weight, crown rump length, brain weight, ratio of brain to liver weight). For viral loads, thyroid hormone levels, and organ weights, fetal phenotyping was not conducted for dead fetuses.

First, fetal viability and survival *in utero* were determined by scoring fetal preservation during necropsy as previously described [24], where fetal preservation scores comprised of: viable (VIA) fetuses, meconium-stained fetuses on head (MEC-H), meconium-stained fetuses on body (MEC-B), decomposed (DEC), autolyzed (AUT), and mummified (MUM) fetuses. Fetal viability compared VIA versus non-VIA (MEC-H, MEC-B, DEC, AUT) fetuses whereas fetal survival compared live (VIA, MEC-H, MEC-B) versus dead (DEC, AUT) fetuses. MUM fetuses were excluded from the analysis since they were presumed to have died before PRRSV-2 inoculation of gilts based on having a crown-rump length (CRL) < 20 cm. Additionally, fetuses were classified using fetal preservation score combined with fetal thymic viral load since this variable (“fetal classification”) was extensively described as a surrogate measure of fetal resilience in response to transplacental PRRSV-2 infection in our previous studies [13, 14, 22, 57]. VIA fetuses were further separated into uninfected (UNIF) with zero log_10_ copies/mg, low viral load (LV-VIA) with < 4 log_10_ copies/mg, or high viral load (HV-VIA) with > 4 log_10_ copies/mg, and MEC-H or MEC-B was merged into a single MEC group (HV-MEC) in the present study. Second, fetal viral loads in serum and thymus were quantified by quantitative reverse transcription-polymerase chain reaction (qRT-PCR) assay, which were expressed by log_10_ transformed PRRSV-2 RNA copies per µL of serum or mg of thymus. Primers and probe were designed based on a genomic sequence (AY545985.1) of PRRSV-2 NVSL 97–7895 and used for detection of a 113 bp length PRRSV-2 RNA between trials as previously described [24]: 5′- TAATGGGCTGGCATTCCT‐3′ (forward primer); 5′‐ ACACGGTCGCCCTAATTG‐3 (reverse primer); 5′-HEX-TGTGGTGAATGGCACTGATTGRCA-BHQ2-3′ (probe). Third, circulating total T4 and T3 concentration (nmol/L) were measured using fetal sera by a solid phase radioimmunoassay with commercially available kits (MP Biomedical, USA) as previously described [13]. Lastly, fetal body, brain, and liver weight (g) were measured at necropsy and used to determine ratio of brain to liver weight. The method used to measure CRL differed between trials in that a measuring tape was used in trial-1 whereas a fetal body image captured from recorded video during necropsy was used in trial-2.

All regression analyses were run in R (v4.1.0) [53]. Univariate mixed-effects linear or logistic regression models were fitted to determine the effect of genotype of Asn91Ser mutation on fetal phenotypes using lme4 (v1.1.27.1) [56] and lmerTest (v3.1.3) [58]. *P* < 0.05 was used to declare a significant association between genotype and fetal outcome, and 0.05 ≤ *P* ≤ 0.10 was considered a trend. Estimated marginal mean (EMM) on fetal phenotypes for each level of genotype were obtained using emmeans (v1.6.2.1) [59] and used for comparing fetal outcomes. Genotype (CC, CD) and trial (trial-1, trial-2) were fixed effects included in all linear or logistic regression models regardless of significance of the coefficients, and fetal preservation (VIA, MEC-H, MEC-B) or fetal classification (UNIF, LV-VIA, HV-VIA, HV-MEC) was included as a fixed effect in all models except logistic regression models. Gilt and experimental batch specific random effects were included in all models. Other variables, such as fetal sex, T4 level, body weight, or litter size, were included to model each fetal outcome. In addition, potential two-way interactions among categorical variables were tested and, if significant, all possible pairwise comparisons among all EMMs for each level of the two interacting factors were computed in post hoc pairwise *t*-tests with *P* values adjusted by the Benjamini-Hochberg (BH) method, using emmeans (v1.6.2.1). Analysis of standardized Pearson residuals was conducted to examine the underlying assumptions of residuals at fetal, gilt, or batch level by plotting residuals against other predictors or fitted values, and by plotting quantile-quantile (Q-Q) plots. If a standardized residual for the individual fetal observation exceeded absolute value of 4, it was excluded from the models, then residual analyses were repeated after refitting models to confirm whether the residual assumptions were still met.

### Cortisol assay analyses

Cortisol concentration (nmol/L) was measured using a radioimmunoassay kit (MP Biomedicals, USA) in sera for a subset of fetuses assessed in association tests. Blood collected from the axillary vessels in fetuses at necropsy was separated and the sera frozen at -80 ℃ until used. The subset of fetuses assessed included 21 CC and 21 CD for trial-1, and 22 CC and 26 CD for trial-2, ensuring a reasonable balance of fetal pigs per genotype within fetal classification. Samples were tested in duplicate. Intra-assay coefficient of variation (CV) was 6.3%, but inter-assay CV was not calculated since all reactions for the assay were run in a single batch.

To explore the relationship among cortisol, T3, and T4 level in fetuses grouped by fetal classification and trials, a scatter plot was created and examined. The association of cortisol level and genotype of Asn91Ser substitution, stratified by trial or fetal classification was tested by approximative two-sample Fisher-Pitman permutation test (two-sample location test) using coin (v1.4-2) [60] in R (v4.2.0) [61]. Additionally, the independence of cortisol level and fetal classification was assessed using approximative K-sample Fisher-Pitman permutation test. Pairwise two-sample permutation tests across groups of fetal classification for multiple comparisons were also conducted using rcompanion (v2.4.18) [62] in R (v4.2.0). Adjusted *P* value by the Benjamini-Hochberg (BH) method was used for multiple comparisons. Statistical significance was defined when *P* value was less than 0.05.

### Electronic supplementary material

Below is the link to the electronic supplementary material.

Additional file 1

Additional file 2

Additional file 3

Additional file 4

Additional file 5

## Data Availability

The genotype and phenotype datasets used and analyzed during the current study are available from the additional file 4 and 5. The sequence datasets generated and analyzed during the current study are available in the Sequence Read Archive (SRA) repository, [Accession: PRJNA1000086].

## Abbreviations

PRRSV: Porcine Reproductive And Respiratory Syndrome Virus
GWAS: Genome Wide Association Study
SNP: Single Nucleotide Polymorphism
SSC7: Sus Scrofa Chromosome 7
DIO2: Iodothyronine Deiodinase 2
Sec: Selenocysteine
T4: Thyroxine (3,5,3′,5′-Tetraiodothyronine)
T3: 3,5,3′-Triiodothyronine
rT3: 3,3′,5′-Triiodothyronine
3,3′-T2: 3,3′-Diiodothyronine
DIO1: Iodothyronine Deiodinase 1
TSHR: Thyroid Stimulating Hormone Receptor
TSH: Thyroid Stimulating Hormone
HPT: Hypothalamic-Pituitary-Thyroid
HPA: Hypothalamic-Pituitary-Adrenal
LD: Linkage Disequilibrium
pCADD: Combined Annotation Dependent Depletion for pig
SECIS: Sec Insertion Sequence
CDKN1A: Cyclin Dependent Kinase Inhibitor 1A
WSB1: WD repeat and SOCS Box-containing protein 1
Thr: Threonine
Ala: Alanine
PRKAG3: Protein Kinase AMP-Activated Non-Catalytic Subunit Gamma 3
PCDH19: Protocadherin 19
ATP: Adenosine Triphosphate
RBP: RNA Binding Protein
miRNA: microRNA
MYH7: Myosin Heavy Chain 7
GBP1: Guanylate Binding Protein 1
SECISBP2: SECIS Binding Protein 2
tRNA^Sec^: Selenocysteyl-tRNA
