# Supplementary material for: A DIO2 missense mutation and its impact on fetal response to PRRSV infection
Source: BMC Vet Res. 2024 Jun 12;20:255. doi: 10.1186/s12917-024-04099-4 (PMC11167750; doi:10.1186/s12917-024-04099-4)
Supplement: Supplementary file 1 — Additional file 1 [file 12917_2024_4099_MOESM1_ESM.pdf]

Supplementary table 1. Single nucleotide polymorphisms (SNPs) identified in pig *DIO2* exons for trial-2 parents.

position	strand	dbSNP ID	SNP label†	Sequence change¶	R^2.with.DRGA SNP‡	*P* value.LD#	pCADD
103096648	-	rs338965211	SNP24	c.*4855G>T	0.045	0.086	0.053
103096741	-	rs80940301	SNP23	c.*4762C>T	0.045	0.086	0.504
103096948	-	rs331231927	SNP22	c.*4555C>T	0.045	0.086	9.568
103096967	-	rs341304760	SNP21	c.*4536G>A	0.832	0.000	0.550
103096995	-	rs323408162	SNP20	c.*4508T>C	0.045	0.086	2.338
103097282	-	rs324131721	SNP19	c.*4221C>T	0.231	0.000	0.057
103099552	-	rs345026587	SNP18	c.*1951C>G	0.409	0.000	0.026
103099651	-	rs324198118	SNP17	c.*1852C>T	0.409	0.000	3.137
103100111	-	rs339409577	SNP16	c.*1392G>T	0.033	0.140	0.026
103100152	-	rs324953755	SNP15	c.*1351C>T	0.885	0.000	0.095
103100321	-	rs326165011	SNP14	c.*1182A>G	0.045	0.086	3.897
103100332	-	rs345554161	SNP13	c.*1171T>G	0.326	0.000	5.388
103100356	-	rs336144847	SNP12	c.*1147T>C	0.209	0.000	1.502
103100401	-	rs318454513	SNP11	c.*1102G>C	0.209	0.000	0.615
103100459	-	rs327964482	SNP10	c.*1044A>G	0.326	0.000	0.140
103100689	-	rs319212422	SNP9	c.*814C>T	0.209	0.000	0.115
103100692	-	rs328655817	SNP8	c.*811G>A	0.209	0.000	2.663
103100763	-	rs345068596	SNP7	c.*740A>G	0.209	0.000	4.295
103100787	-	rs320872938	SNP6	c.*716T>G	0.209	0.000	0.035
103101248	-	rs338724710	SNP5	c.*255T>C	0.301	0.000	10.244
103101389	-	rs324974389	SNP4	c.*114G>C	0.21	0.000	0.775
103101455	-	rs340742293	SNP3	c.*48G>A	0.016	0.304	0.288
103102040	-	rs323844025	SNP2	c.273T>C	0.045	0.086	0.442
103102041	-	rs333347361	SNP1	c.272A>G	0.045	0.086	0.001

Parental DNA samples (N=33, 27 gilts, 6 sires) from trial-2 [1] were used to detect SNPs.

†SNP label is the same as the labels of SNP in the heatmap of genotypes and haplotypes across *DIO2* SNPs as shown in Figure 1B and 1C.

[¶]Sequence change follows sequence variant nomenclature by Human Genome Variation Society (HGVS): e.g., c*4855G>T denotes a substitution of the G nucleotide by a T at base position 4855 from the 3' of the translation stop codon in the coding DNA (cDNA) reference sequence. c.272A>G denotes a substitution of the A nucleotide by a G at base position 272 from A of the ATG-translation initiation codon in the cDNA sequence.

[‡]R^2.with.DRGA SNP, Linkage disequilibrium (LD) estimate using squared allelic correlation coefficient (R^2) between DRGA0008048 and each SNP detected.

[#]*P* value.LD, significance of LD estimate.

Supplementary table 2. Known or proposed causative variants in pigs annotated by pCADD scores.

Gene (variant)	Chromosomal locus	Variant effect	dbSNP ID	Trait affected	pCADD score	Reference
MC4R (p.Asp298Asn)	1:160,773,437	Missense variant	rs81219178	Growth and fatness	27.477	[2]
NR6A1 (p.Leu192Pro)	1:265347265	Missense variant	rs326780270	Vertebrae number	17.198	[3]
IGF2 (g.1483817T>C)	2:1,483,817	Intronic variant		Muscle growth	15.609	[4]
PHKG1 (g.16830320C>A)	3:16,830,320	Splice region variant	rs330928088	Glycogen content and meat quality	2.130	[5]
POLR1B (g.43952776T>G)	3:43952776	splice region variant		Lethal recessives	10.144	[6]
RYR1 (p.Arg651Cys)	6:47,357,966	Missense variant	rs344435545	Malignant hypothermia	0.139	[7, 8]
LEPR (p.Leu663Phe)	6:146829589	Missense variant	rs709596309	Productive, fatness and meat quality	22.868	[9]
PNKP (p.Gln96Arg)	6:54880241	Missense variant		Lethal recessives	28.767	[6]
VRTN (g.97614602A>C)	7:97,614,602	Noncoding variant	rs709317845	Vertebrae number	11.952	[10]
PPARD (p.Gly32Glu)	7:31281804	Missense variant	rs80909573	Ear size, fat metabolism, skin and cartilage development	21.589	[11]
SYNGR2 (p.Cys63Arg)	12:3,797,515	Missense variant	rs3473454700	Porcine circovirus viral load	0.00013	[12]
TADA2A (g.38922102G>A)	12:38922102	splice donor variant		Lethal recessives	21.848	[6]
MSTN (p.Glu274*)	15:94,623,834	Stop gain variant		Leg weakness	38.959	[13]
PRKAG3 (p.Arg250Gln)	15:120,863,533	Missense variant	rs1109104772	Glycogen content and meat quality	32.657	[14]
PCK1 (p.Met139Leu)	17:57932233	Missense variant	rs343196765	Intramuscular fat content, backfat thickness and meat quality	23.322	[15]

This table was based on published tables for known or proposed causal variants in pigs [16, 17].

Supplementary table 3. Comparison of fetal phenotypes grouped by level of log fold change in *DIO2* expression in fetal heart.

Fetal genotype and phenotype¶	Group by log FC of *DIO2* High† (n=5)	Mid† (n=9)	Mid (n=2)
Asn91Ser genotype	CD	CC	CD
logddDIO2	1.7 (0.4)	-0.2 (0.3)	0 (0.7)
Brain to liver weight	1.4 (0.1)	1.3 (0.1)	1.3 (0.1)
**Viral load in placenta**	**5.3 (0.6)**	**3.7 (1.3)**	**1.7 (1.7)**
**Viral load in serum**	**7.6 (0.6)**	**4.1 (2.6)**	**1.7 (0.6)**
**Viral load in thymus**	**6.8 (0.3)**	**3.5 (2)**	**1.6 (0.7)**
**T4 (nmol/L)**	**54.6 (15.5)**	**70.9 (21.9)**	**75.6 (4.1)**
T3 (nmol/L)	0.6 (0.1)	0.6 (0.2)	0.6 (0.1)
litter size	17.6 (3.3)	17.3 (2.3)	20 (0)
Fetal weight (g)	930.6 (87.6)	876.6 (126.1)	932 (39.4)
Crown rump length (cm)	36.9 (1.2)	35.6 (1.7)	37.1 (0.1)
Girth (cm)	8.6 (0.4)	8.4 (0.5)	8.7 (0)
Brain weight (g)	28.7 (1.3)	25.9 (2.4)	29.4 (1.8)
Liver weight (g)	21.2 (2.1)	20.2 (1.9)	22.2 (0.8)
Heart weight (g)	6.8 (0.6)	7.1 (1.2)	7.8 (0.2)
Lung weight (g)	25.4 (3.2)	26.4 (4.9)	28.9 (0.2)
Thyroid weight (mg)	213 (41.9)	204.9 (55.2)	243.5 (38.9)
Adrenal weight (mg)	216.8 (49.4)	146.7 (46.4)	146.5 (53)
Spleen weight (g)	1.5 (0.2)	1.3 (0.3)	1.4 (0.3)

¶Fetal phenotypes were represented by mean (SD); phenotypes were bolded if high group showed a deviation from mid groups.

†5 CD fetuses with log fold change (FC) of *DIO2* >1 (High) were compared with 9 CC or 2 CD fetuses with -0.5 ≤ log FC ≤ 0.5 (Mid), while being at the same range of the covariate (1.1 ≤ brain to liver weight ≤ 1.46).

Supplementary table 4. Estimated marginal mean (SE or 95% CI) associating Asn91ser genotypes (CC, CD) with fetal outcomes.

Fetal outcomes	Estimated marginal mean (SE or 95% CI)						Other predictors included
	Trial-1			Trial-2			
	CC	CD	*P value*	CC	CD	*P value*	
Fetal viability[†]	0.66 (0.41-0.84)	0.57 (0.32-0.78)	0.54	0.41 (0.22-0.63)	0.75 (0.54-0.88)	0.18	sex
Fetal survival[‡]	0.64 (0.39-0.83)	0.73 (0.48-0.88)	0.33	0.73 (0.55-0.86)	0.81 (0.65-0.90)	0.33	sex
**Viral loads**							
Serum viral loads (log10 copies/ul)	2.18 (1.36)	1.58 (1.37)	0.21	5.75 (0.93)	5.16 (0.93)	0.21	fetal preservation, T4 level
Thymic viral loads (log10 copies/mg)	2.48 (1.45)	1.78 (1.46)	0.15	4.43 (1.00)	3.73 (0.99)	0.15	fetal preservation, T4 level
**Thyroid hormone levels**							
T4 in serum (nmol/L)	25.23 (2.70)	21.47 (2.86)	0.16	77.11 (2.14)	73.35 (2.09)	0.16	fetal classification, litter size, sex
T3 in serum (nmol/L)	0.59 (0.08)	0.61 (0.08)	0.64	0.75 (0.06)	0.77 (0.06)	0.64	fetal classification, body weight
**Morphometrics**							
Body weight (g)[¶]	816.17 (43.60)	900.73 (44.87)	0.13	996.39 (39.11)	875.89 (38.75)	0.13	fetal classification, litter size
Crown rump length (cm)[¶]	26.68 (0.60)	28.15 (0.62)	0.009	37.03 (0.56)	35.11 (0.56)	0.031	fetal classification, litter size
**Organ weights**							
Brain (g)	24.15 (0.60)	24.13 (0.61)	0.98	26.87 (0.46)	26.86 (0.46)	0.98	fetal classification
Ratio: brain to liver weight	1.08 (0.09)	1.08 (0.09)	0.9	1.11 (0.07)	1.10 (0.07)	0.9	fetal classification, litter size

[†]Estimated probability (95% CI) indicated for interaction effect between Asn91Ser and trial, with *P* value adjusted by Benjamini-Hochberg (BH) method across all possible pairwise comparisons.

[‡]Estimated probability (95% CI)

[¶]Estimated marginal mean (SE) indicated for interaction effect between Asn91Ser and trial, with *P* value adjusted by Benjamini-Hochberg (BH) method across all possible pairwise comparisons.

Supplementary table 5. Determination of sequence variants in pig *TSHR* region for trial-2 sires (N=6).

position	strand	dbSNP ID	SNP label	sequence change	R^2.with.Asn91Ser[†]	*P*.value.LD[‡]
103768370	+	no dbSNP	5_prime_UTR_variant1	c.-619T>C	0.06642523	0.37196101
103768497	+	no dbSNP	5_prime_UTR_variant2	c.-492C>T	0.06642523	0.37196101
103768500	+	no dbSNP	5_prime_UTR_variant3	c.-489T>C	0.46621872	0.018015671
103768553	+	no dbSNP	5_prime_UTR_variant4	c.-436C>T	0.06642523	0.37196101
103768590	+	no dbSNP	5_prime_UTR_variant5	c.-399T>C	0.46621872	0.018015671
103768716	+	no dbSNP	5_prime_UTR_variant6	c.-273T>C	0.46621872	0.018015671
103768817	+	no dbSNP	5_prime_UTR_variant7	c.-172A>C	0.199701409	0.206242057
103768826	+	no dbSNP	5_prime_UTR_variant8	c.-163G>A	0.110862332	0.346319129
103768890	+	no dbSNP	5_prime_UTR_variant9	c.-99T>G	0.199701409	0.206242057
103768892	+	no dbSNP	5_prime_UTR_variant10	c.-97_-96insCGAGT	NA[¶]	NA
103768930	+	no dbSNP	5_prime_UTR_variant11	c.-59A>G	0.237775307	0.09118638
103847623	+	rs1112876687	intron_variant1	c.171-145G>C	0.46621872	0.018015671
103847991	+	rs1111127688	intron_variant2	c.242+152C>T	0.46621872	0.018015671
103853698	+	rs1107841267	intron_variant3	c.317+113_317+114insCT	NA	NA
103867986	+	rs1108767362	intron_variant4	c.318-122G>A	0.666106717	0.004695127
103868013	+	rs1109465473	intron_variant5	c.318-95A>G	0.666106717	0.004695127
103868217	+	No dbSNP	intron_variant6	c.392+35_392+40insB[#]	0.666106717	0.004695127
103874397	+	rs792895037	intron_variant7	c.468-9C>T	0.030134093	0.54761393
103874498	+	rs1107939652	intron_variant8	c.545+15T>C	0.110862332	0.248743321
103877467	+	rs1112574587	intron_variant9	c.546-505A>G	0.46621872	0.018015671
103877514	+	No dbSNP	intron_variant10	c.546-458A>G	0.46621872	0.018015671
103877580	+	No dbSNP	intron_variant11	c.546-392C>A	0.46621872	0.018015671

103877650	+	rs1110746575	intron_variant12	c.546-322G>A	0.46621872	0.018015671
103877694	+	rs1112391719	intron_variant13	c.546-278T>C	0.46621872	0.018015671
103877788	+	rs1113090843	intron_variant14	c.546-184A>C	0.46621872	0.018015671
103877791	+	rs1110448239	intron_variant15	c.546-181G>T	0.46621872	0.018015671
103877804	+	rs1111217740	intron_variant16	c.546-168T>C	0.46621872	0.018015671
103877955	+	rs1110230512	intron_variant17	c.546-17A>T	0.46621872	0.018015671
103889483	+	rs1109842851	intron_variant18	c.615-178A>C	0.46621872	0.018015671
103923199	+	rs699096214	missense_variant	c.1618A>G	NA	NA
103922622	+	rs196952307	synonymous_variant1	p.His347=	0.46621872	0.018015671
103922631	+	rs196957158	synonymous_variant2	p.Ser350=	0.46621872	0.018015671
103924033	+	rs1111686982	3_prime_UTR_variant1	c.*157G>A	0.46621872	0.018015671

[†]R^2.with.Asn91Ser: Linkage disequilibrium (LD) estimate using squared allelic correlation coefficient (R^2) between the coding variant (p.Asn91Ser) in *DIO2* and each SNP detected.

[¶]NA, LD not estimated since all sires had the same genotype, just differing compared to reference sequence.

[‡]*P* value.LD, significance of LD estimate

[#]B allele denotes inserted alleles collectively compared to reference sequence at SSC7:1038682

Supplementary table 6. Primers used for Sanger sequencing.

Gene name (Ensembl Gene stable ID[†])	Amplicons	Forward primer (5'→3')	Reverse primer (5'→3')	Amplicon length (bp)	Overlap with previous amplicon	Overlap with next amplicon
DIO2 (ENSSSCG00000040638)[‡]	amplicon_1	GGCTGGAGAGACTGGACTTG	AGATGGTTCTGCTGCCAACT	1108	-	-
	amplicon_2A	CCATGATGGCTCTTTCCTCA	GGGCTCTATCCATGCTGAAG	1177	-	244
	amplicon_2B	GCTCAAAAGTAGCCCCATCA	GCCTTACATCAAAGCCTCCA	1150	244	443
	amplicon_2C	GAGAATGGCAGATGGAGAGG	TGGAACAAAGGGGAAGTTTG	1295	443	404
	amplicon_2D	TCAGTGTGCAAGAACCAAAAG	ATTCATGCCCATTCAGGAAA	1268	404	355
	amplicon_2E	TTTCAATATCCACCCCACCT	GGGACAGAAGTTGGTGCCTA	1007	355	276
	amplicon_2F	CTGCCTTGCTGCATAAAACA	AGAGCTGCTGCCCAAGATAG	944	276	351
	amplicon_2G	GAACAGGACCTGGGAGATCA	CCAGGGGCAAGTTCTAGAGAG	1112	351	-
TSHR (ENSSSCG00000031771)[¶]	amplicon_1	GGCAGACCAGAATTTACCAGTTG	GGCCTCTTCTCAAGCTCTTTTTG	1124	-	-
	amplicon_2	CATCCCAGTCTACTGCTACTTCAG	ACCCGTCGTGAAAAGAGAATCAA	701	-	-
	amplicon_3	AGGACTAATGTAGTGGAGGAACT	GCTGACACGGTAGTACAGAAAG	813	-	-
	amplicon_4	AATCCACTCAAACCTCCACTC	AGTTCCCATCGTGGTTCAATAG	764	-	-
	amplicon_5	CCATGGTCTCTCAGGTAATTAAGG	TTCTTTCTTGAGCCCACTCC	505	-	-
	amplicon_6	TGAAATCCATTGGTCCCATTCT	GACTTTGGGACGGTCACTTAAA	963	-	-
	amplicon_7	CCATGGTCTGTCTGCTTTCT	GGTCGTACTAACTCTGGTGTTG	759	-	-
	amplicon_8	TGCCATTGGCCAGGTAAG	CCAACTATTCACACTCCTCCTTT	748	-	-
	amplicon_9	ACAGGAGTGGGAGCAATTTAG	GCAAGATCTGGTCATCTCCTAAA	856	-	-
	amplicon_10	CTTGAATTGCTTGCAGATGAGAA	GTCTTTGTCTCCCGGGTTATAC	1198	-	418
	amplicon_11	GTACTACAACCATGCCATCGAC	GCCATTGTTGCATTTAGCATCT	1100	418	-

[†]Ensembl gene ID for Pig genes (Sscrofa11.1) (Ensembl Release 105 (Dec 2021) used).

[‡]For *DIO2*, the first coding exon (ENSSSCE00000310588; 275 bp length;) was covered by 1 amplicon (amplicon_1) and the second coding exon (ENSSSCE00000324921; 5666 bp length) covered by 7 amplicons (amplicon 2A to 2G).

[¶]For *TSHR*, a predicted transcript isoform, TSHR-202 (ENSSSCT00000038969.2), was targeted for sequencing. Region of 1st (ENSSSCE00000332089) to 2nd (ENSSSCE00000288966) exon including 1st intron (863 bp length) covered by amplicon_1; translated region (1414 bp length) of 11th exon (ENSSSCE00000324742) covered by 2

amplicons, amplicon_10 and 11. The rest of exons (3rd to 10th exon) covered by each amplicon (amplicon_2 to amplicon_9).

Annealing temperature for all amplicons was 60℃.

Supplementary table 7. Primers used for quantitative real-time PCR.

Gene name (Ensembl Gene stable ID†)	Forward primer (5'→3')	Reverse primer (5'→3')	Annealing temp (°C)	Amplicon length (bp)
DIO2 (ENSSSCG00000040638)	CTCGGTCATTCTCCTCAAGC	TCACCTGTTTGTAGGCATCG	61	140
ACTB (ENSSSCG00000007585)	CCAGCACGATGAAGATCAAG	AGTCCGCCTAGAAGCATTTG	60	171
HMBS (ENSSSCG00000015108)	AGGATGGGCAACTCTACCTG	GATGGTGGCCTGCATAGTCT-	61	83
SDHA (ENSSSCG00000020686)	CTACAAGGGGCAGGTTCTGA	AAGACAACGAGGTCCAGGAG	61	141
STX5 (ENSSSCG00000026293)	TGCAGAGTCGTCAGAATGGA	CCAGGATTGTCAGCTTCTCC	60	144

†Ensembl gene ID for Pig genes (Sscrofa11.1) (Ensembl Release 105 (Dec 2021) used).

Supplementary table 8. Selected fetal population for association analysis.

Genotyping method	Trials	
	trial-1†	trial-2†
TaqMan assay	78	59
inferred by parental genotypes from Sanger sequencing	0	86
Total	78	145

†trial-1, GWAS of Yang et al.(2016) [18]; trial-2, follow up study of Ko et al. (2022) [1].

References

1. Ko H, Sammons J, Pasternak JA, Hamonic G, Starrak G, MacPhee DJ, Detmer SE, Plastow GS, Harding JCS. Phenotypic effect of a single nucleotide polymorphism on SSC7 on fetal outcomes in PRRSV-2 infected gilts. Livestock Science. 2022;255:104800.
2. Kim KS, Larsen N, Short T, Plastow G, Rothschild MF. A missense variant of the porcine melanocortin-4 receptor (MC4R) gene is associated with fatness, growth, and feed intake traits. Mammalian Genome. 2000;11(2):131-5.
3. Fontanesi L, Ribani A, Scotti E, Utzeri VJ, Veličković N, Dall'Olio S. Differentiation of meat from European wild boars and domestic pigs using polymorphisms in the MC1R and NR6A1 genes. Meat Science. 2014;98(4):781-4.
4. Van Laere A-S, Nguyen M, Braunschweig M, Nezer C, Collette C, Moreau L, Archibald AL, Haley CS, Buys N, Tally M, Andersson G, Georges M, Andersson L. A regulatory mutation in IGF2 causes a major QTL effect on muscle growth in the pig. Nature. 2003;425(6960):832-6.
5. Ma J, Yang J, Zhou L, Ren J, Liu X, Zhang H, Yang B, Zhang Z, Ma H, Xie X, Xing Y, Guo Y, Huang L. A splice mutation in the PHKG1 gene causes high glycogen content and low meat quality in pig skeletal muscle. Plos Genetics. 2014;10(10):e1004710.
6. Derks MFL, Gjuvsland AB, Bosse M, Lopes MS, Van Son M, Harlizius B, Tan BF, Hamland H, Grindflek E, Groenen MAM, Megens H-J. Loss of function mutations in essential genes cause embryonic lethality in pigs. Plos Genetics. 2019;15(3):e1008055.
7. Fujii J, Otsu K, Zorzato F, De Leon S, Khanna VK, Weiler JE, O'Brien PJ, MacLennan DH. Identification of a mutation in porcine ryanodine receptor associated with malignant hyperthermia. Science. 1991;253(5018):448-51.
8. Otsu K, Khanna VK, Archibald AL, Maclennan DH. Cosegregation of porcine malignant hyperthermia and a probable causal mutation in the skeletal muscle ryanodine receptor gene in backcross families. Genomics. 1991;11(3):744-50.
9. Óvilo C, Fernández A, Fernández AI, Folch JM, Varona L, Benítez R, Nuñez Y, Rodríguez C, Silió L. Hypothalamic expression of porcine leptin receptor (LEPR), neuropeptide Y (NPY), and cocaine- and amphetamine-regulated transcript (CART) genes is influenced by LEPR genotype. Mammalian Genome. 2010;21(11-12):583-91.
10. Fan Y, Xing Y, Zhang Z, Ai H, Ouyang Z, Ouyang J, Yang M, Li P, Chen Y, Gao J, Li L, Huang L, Ren J. A further look at porcine chromosome 7 reveals VRTN variants associated with vertebral number in Chinese and Western pigs. PloS One. 2013;8(4):e62534.
11. Ren J, Duan Y, Qiao R, Yao F, Zhang Z, Yang B, Guo Y, Xiao S, Wei R, Ouyang Z, Ding N, Ai H, Huang L. A missense mutation in PPARD causes a major QTL effect on ear size in pigs. Plos Genetics. 2011;7(5):e1002043.
12. Walker LR, Engle TB, Vu H, Tosky ER, Nonneman DJ, Smith TPL, Borza T, Burkey TE, Plastow GS, Kachman SD, Ciobanu DC. Synaptogyrin-2 influences replication of Porcine circovirus 2. Plos Genetics. 2018;14(10):e1007750.

13. Matika O, Robledo D, Pong-Wong R, Bishop SC, Riggio V, Finlayson H, Lowe NR, Hoste AE, Walling GA, Del-Pozo J, Archibald AL, Woolliams JA, Houston RD. Balancing selection at a premature stop mutation in the myostatin gene underlies a recessive leg weakness syndrome in pigs. Plos Genetics. 2019;15(1):e1007759.
14. Milan D, Jeon J-T, Looft C, Amarger V, Robic A, Thelander M, Rogel-Gaillard C, Paul S, Iannuccelli N, Rask L, Ronne H, Lundström K, Reinsch N, Gellin J, Kalm E, Roy Pascale L, Chardon P, Andersson L. A mutation in PRKAG3 associated with excess glycogen content in pig skeletal muscle. Science. 2000;288(5469):1248-51.
15. Latorre P, Burgos C, Hidalgo J, Varona L, Carrodeguas JA, López-Buesa P. c.A2456C-substitution in Pck1 changes the enzyme kinetic and functional properties modifying fat distribution in pigs. Scientific Reports. 2016;6(1):19617.
16. Groß C, Derks M, Megens H-J, Bosse M, Groenen MAM, Reinders M, De Ridder D. pCADD: SNV prioritisation in *Sus scrofa*. Genetics Selection Evolution. 2020;52(1):1-15.
17. Johnsson M, Jungnickel MK. Evidence for and localization of proposed causative variants in cattle and pig genomes. Genetics Selection Evolution. 2021;53(1):67.
18. Yang T, Wilkinson J, Wang Z, Ladinig A, Harding J, Plastow G. A genome-wide association study of fetal response to type 2 porcine reproductive and respiratory syndrome virus challenge. Scientific Reports. 2016;6(1):20305.
