# Supplementary material for: A DIO2 missense mutation and its impact on fetal response to PRRSV infection
Source: BMC Vet Res. 2024 Jun 12;20:255. doi: 10.1186/s12917-024-04099-4 (PMC11167750; doi:10.1186/s12917-024-04099-4)
Supplement: Supplementary file 2 — Additional file 2 [file 12917_2024_4099_MOESM2_ESM.pdf]

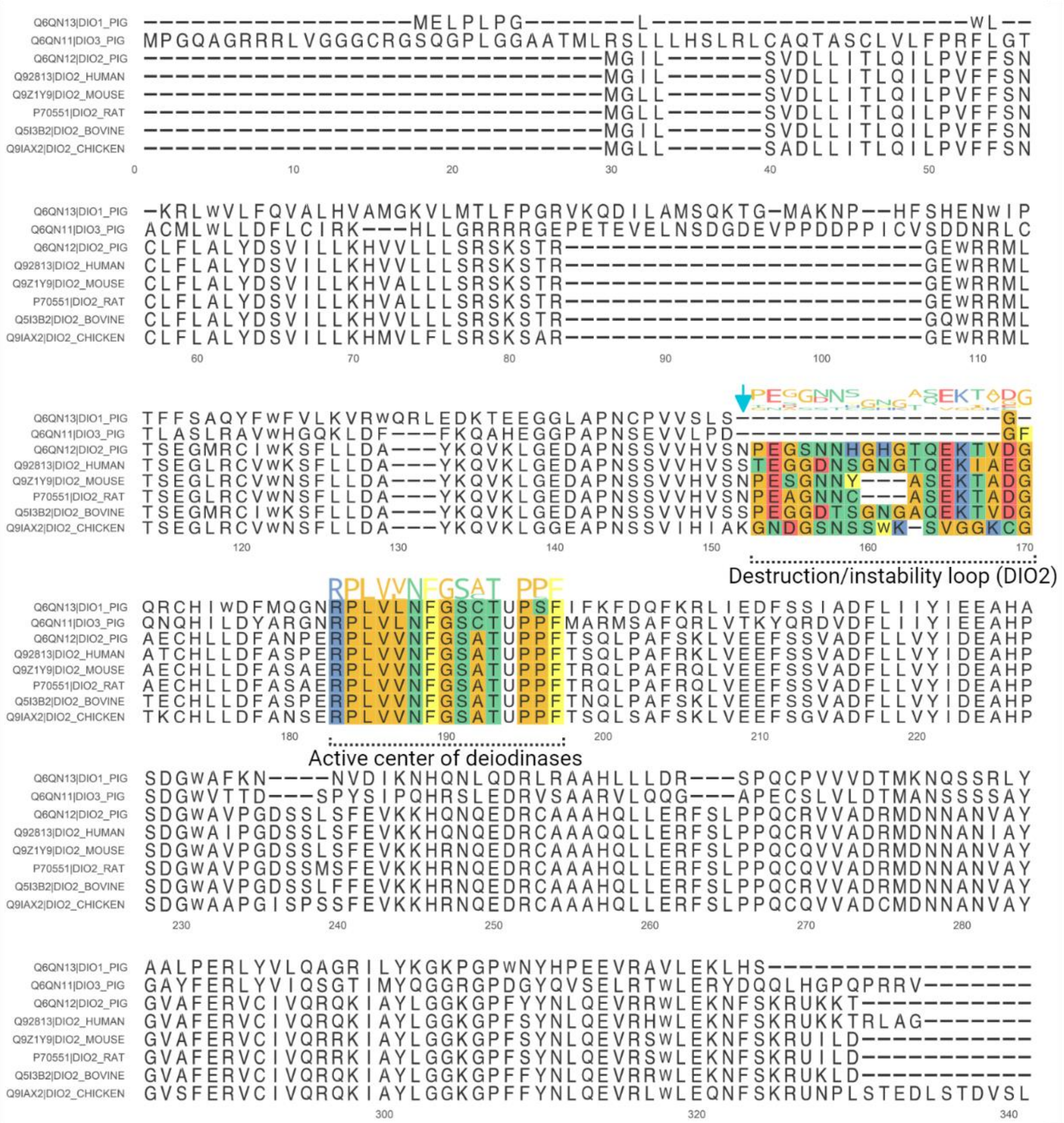

Additional file 2. Multiple protein sequence alignment across deiodinases of pigs and other species.

Protein sequences of porcine deiodinases (DIO1, DIO2, DIO3) were aligned with DIO2 sequences from human, mouse, rat, cattle, and chicken via the web interface for Clustal Omega (https://www.ebi.ac.uk/Tools/msa/clustalo/) with the default settings, and aligned result was plotted using ggmsa [1] (v.1.0.0) in R. Light blue arrow denotes amino acid position altered by Asn91Ser variant. Destruction (or instability) loop [2, 3] and active center of deiodinases [4] were annotated by color coded amino acids and sequence logo. UniProt ID for each protein sequence is on the left side of the vertical bar for the sequence label. Edited with Biorender.

References

1. Zhou L, Li L, Dai Z, Yu G, Feng T, Guan Y, Xu S, Gao F, Lam TT, Wang Q, Wu T, Huang H, Zhan L. Ggmsa: A visual exploration tool for multiple sequence alignment and associated data. Briefings in Bioinformatics. 2022;23(4).
2. Dentice M, Bandyopadhyay A, Gereben B, Callebaut I, Christoffolete MA, Kim BW, Nissim S, Mornon J-P, Zavacki AM, Zeöld A, Capelo LP, Curcio-Morelli C, Ribeiro R, Harney JW, Tabin CJ, Bianco AC. The Hedgehog-inducible ubiquitin ligase subunit WSB-1 modulates thyroid hormone activation and PTHrP secretion in the developing growth plate. Nature Cell Biology. 2005;7(7):698-705.
3. Zeo¨ld A, Pormu¨ller Lv, Dentice M, Harney JW, Curcio-Morelli C, Tente SM, Bianco AC, Gereben Bz. Metabolic instability of type 2 deiodinase is transferable to stable proteins independently of subcellular localization. Journal of Biological Chemistry. 2006;281(42):31538-43.
4. Bianco AC, Salvatore D, Gereben B, Berry MJ, Larsen PR. Biochemistry, cellular and molecular biology, and physiological roles of the iodothyronine selenodeiodinases. Endocrine Reviews. 2002;23(1):38-89.
