# Supplementary material for: A DIO2 missense mutation and its impact on fetal response to PRRSV infection
Source: BMC Vet Res. 2024 Jun 12;20:255. doi: 10.1186/s12917-024-04099-4 (PMC11167750; doi:10.1186/s12917-024-04099-4)
Supplement: Supplementary file 3 — Additional file 3 [file 12917_2024_4099_MOESM3_ESM.pdf]

PRRSV dysregulates fetal hypothalamic-pituitary-adrenal/thyroid axis independently of a missense mutation in the DIO2 gene. Ko *et al.*

ARRIVE 10 guidelines 2.0

		Section/line number, or reason for not reporting	
		Trial	
		trial-1	trial-2
1. Study design	a. The groups being compared, including control groups. If no control group has been used, the rationale should be stated.	See gene expression analysis, phenotypic association analyses, cortisol assay analyses in the materials and methods	See gene expression analysis, phenotypic association analyses, cortisol assay analyses in the materials and methods
	b. The experimental unit (e.g. a single animal, litter, or cage of animals).	See PRRSV challenge experiments and collection of fetal phenotypes in the materials and methods	See PRRSV challenge experiments and collection of fetal phenotypes in the materials and methods
2. Sample size	a. Specify the exact number of experimental units allocated to each group, and the total number in each experiment. Also indicate the total number of animals used.	See legends in figure 2-figure 5 and supplementary table 8 in additional file 1	See legends in figure 2-figure 5 and supplementary table 8 in additional file 1
	b. Explain how the sample size was decided. Provide details of any a priori sample size calculation, if done.	See fetal Asn91Ser genotyping in the materials and methods	See fetal Asn91Ser genotyping in the materials and methods
3. Inclusion and exclusion criteria	a. Describe any criteria used for including and excluding animals (or experimental units) during the experiment, and data points during the analysis. Specity if these criteria were established a priori. If no criteria were set, state this explicitly.	NA	NA
	b. For each experimental group, report any animals, experimental units or data points not included in the analysis and explain why. If there were no exclusions, state so.	See regression analysis in the materials and methods	See regression analysis in the materials and methods
	c. For each analysis, report the exact value of n in each experimental group.	See legends in figure 2-figure 5 and supplementary table 8 in additional file 1	See legends in figure 2-figure 5 and supplementary table 8 in additional file 1
4. Randomisation	a. State whether randomisation was used to allocate experimental units to control and treatment groups. If done, provide the method used to generate the randomisation sequence.	NA	NA
	b. Describe the strategy used to minimise potential confounders such as the order of treatments and measurements, or animal/cage location. If confounders were not controlled, state this explicitly.	NA	NA
5. Blinding	Describe who was aware of the group allocation at the different stages of the experiment (during the allocation, the conduct of the experiment, the outcome assessment, and the data analysis).	NA	NA
6. Outcome measures	a. Clearly define all outcome measures assessed (e.g. cell death, molecular markers, or behavioural changes).	See gene expression analysis, phenotypic association analyses,	See gene expression analysis, phenotypic association analyses,
	b. For hypothesis-testing studies, specify the primary outcome measure, i.e. the outcome measure that was used to determine the sample size.	NA	
7. Statistical methods	a. Provide details of the statistical methods used for each analysis, including software used.	See gene expression analysis, regression analysis, cortisol assay analyses in the materials and methods	See gene expression analysis, regression analysis, cortisol assay analyses in the materials and methods
	b. Describe any methods used to assess whether the data met the assumptions of the statistical approach, and what was done if the assumptions were not met.	See regression analysis in the materials and methods	See regression analysis in the materials and methods
8. Experimental animals	a. Provide species-appropiate details of the animals used, including species, strain and substrain, sex, age or developmental stage, and, if relevant, weight.	See PRRSV challenge experiments and collection of fetal phenotypes in the materials and methods	See PRRSV challenge experiments and collection of fetal phenotypes in the materials and methods
	b. Provide further relevant information on the provenance of animals, health/immune status, genetic modification status, genotype, and any previous procedures.	See PRRSV challenge experiments and collection of fetal phenotypes in the materials and methods	See PRRSV challenge experiments and collection of fetal phenotypes in the materials and methods
9. Experimental procedures	For each experimental group, including controls, describe the procedures in enough detail to allow others to replicate them, including:		
	a. What was done, how it was done and what was used.	See all sections in the materials and methods	See all sections in the materials and methods
	b. When and how often.		
	c. Where (including detail of any acclimatisation periods).		
	d. Why (provide rationale for procedures).		
10. Results	For each experiment conducted, including independent replications, report:		
	a. Summary/descriptive statistics for each experimental group, with a measure of variability where applicable (e.g. mean and SD, or median and range).	NA	
	b. If applicable, the effect size with a confidence interval	NA	
