# Supplementary material for: A DIO2 missense mutation and its impact on fetal response to PRRSV infection
Source: BMC Vet Res. 2024 Jun 12;20:255. doi: 10.1186/s12917-024-04099-4 (PMC11167750; doi:10.1186/s12917-024-04099-4)
Supplement: Supplementary file 5 — Additional file 5 [file 12917_2024_4099_MOESM5_ESM.docx]

**Supplementary table 1.** Single nucleotide polymorphisms (SNPs) identified in pig *DIO2* exons for trial-2 parents.

| position | strand | dbSNP ID | SNP label^†^ | Sequence change^¶^ | R^2^.with.DRGA SNP^ǂ^ | *P* value.LD^#^ | pCADD |
| --- | --- | --- | --- | --- | --- | --- | --- |
| 103096648 | - | rs338965211 | SNP24 | c.*4855G>T | 0.045 | 0.086 | 0.053 |
| 103096741 | - | rs80940301 | SNP23 | c.*4762C>T | 0.045 | 0.086 | 0.504 |
| 103096948 | - | rs331231927 | SNP22 | c.*4555C>T | 0.045 | 0.086 | 9.568 |
| 103096967 | - | rs341304760 | SNP21 | c.*4536G>A | 0.832 | 0.000 | 0.550 |
| 103096995 | - | rs323408162 | SNP20 | c.*4508T>C | 0.045 | 0.086 | 2.338 |
| 103097282 | - | rs324131721 | SNP19 | c.*4221C>T | 0.231 | 0.000 | 0.057 |
| 103099552 | - | rs345026587 | SNP18 | c.*1951C>G | 0.409 | 0.000 | 0.026 |
| 103099651 | - | rs324198118 | SNP17 | c.*1852C>T | 0.409 | 0.000 | 3.137 |
| 103100111 | - | rs339409577 | SNP16 | c.*1392G>T | 0.033 | 0.140 | 0.026 |
| 103100152 | - | rs324953755 | SNP15 | c.*1351C>T | 0.885 | 0.000 | 0.095 |
| 103100321 | - | rs326165011 | SNP14 | c.*1182A>G | 0.045 | 0.086 | 3.897 |
| 103100332 | - | rs345554161 | SNP13 | c.*1171T>G | 0.326 | 0.000 | 5.388 |
| 103100356 | - | rs336144847 | SNP12 | c.*1147T>C | 0.209 | 0.000 | 1.502 |
| 103100401 | - | rs318454513 | SNP11 | c.*1102G>C | 0.209 | 0.000 | 0.615 |
| 103100459 | - | rs327964482 | SNP10 | c.*1044A>G | 0.326 | 0.000 | 0.140 |
| 103100689 | - | rs319212422 | SNP9 | c.*814C>T | 0.209 | 0.000 | 0.115 |
| 103100692 | - | rs328655817 | SNP8 | c.*811G>A | 0.209 | 0.000 | 2.663 |
| 103100763 | - | rs345068596 | SNP7 | c.*740A>G | 0.209 | 0.000 | 4.295 |
| 103100787 | - | rs320872938 | SNP6 | c.*716T>G | 0.209 | 0.000 | 0.035 |
| 103101248 | - | rs338724710 | SNP5 | c.*255T>C | 0.301 | 0.000 | 10.244 |
| 103101389 | - | rs324974389 | SNP4 | c.*114G>C | 0.21 | 0.000 | 0.775 |
| 103101455 | - | rs340742293 | SNP3 | c.*48G>A | 0.016 | 0.304 | 0.288 |
| 103102040 | - | rs323844025 | SNP2 | c.273T>C | 0.045 | 0.086 | 0.442 |
| 103102041 | - | rs333347361 | SNP1 | c.272A>G | 0.045 | 0.086 | 0.001 |

Parental DNA samples (N=33, 27 gilts, 6 sires) from trial-2 [1] were used to detect SNPs.

^†^SNP label is the same as the labels of SNP in the heatmap of genotypes and haplotypes across *DIO2* SNPs as shown in Figure 1B and 1C.

^¶^Sequence change follows sequence variant nomenclature by Human Genome Variation Society (HGVS): e.g., c*4855G>T denotes a substitution of the G nucleotide by a T at base position 4855 from the 3' of the translation stop codon in the coding DNA (cDNA) reference sequence. c.272A>G denotes a substitution of the A nucleotide by a G at base position 272 from A of the ATG-translation initiation codon in the cDNA sequence.

^ǂ^R^2^.with.DRGA SNP, Linkage disequilibrium (LD) estimate using squared allelic correlation coefficient (R^2^) between DRGA0008048 and each SNP detected.

^#^*P* value.LD, significance of LD estimate.

**Supplementary table 2.** Known or proposed causative variants in pigs annotated by pCADD scores.

| Gene (variant) | Chromosomal locus | Variant effect | dbSNP ID | Trait affected | pCADD score | Reference |
| --- | --- | --- | --- | --- | --- | --- |
| MC4R (p.Asp298Asn) | 1:160,773,437 | Missense variant | rs81219178 | Growth and fatness | 27.477 | [2] |
| NR6A1 (p.Leu192Pro) | 1:265347265 | Missense variant | rs326780270 | Vertebrae number | 17.198 | [3] |
| IGF2 (g.1483817T>C) | 2:1,483,817 | Intronic variant |  | Muscle growth | 15.609 | [4] |
| PHKG1 (g.16830320C>A) | 3:16,830,320 | Splice region variant | rs330928088 | Glycogen content and meat quality | 2.130 | [5] |
| POLR1B (g.43952776T>G) | 3:43952776 | splice region variant |  | Lethal recessives | 10.144 | [6] |
| RYR1 (p.Arg651Cys) | 6:47,357,966 | Missense variant | rs344435545 | Malignant hypothermia | 0.139 | [7, 8] |
| LEPR (p.Leu663Phe) | 6:146829589 | Missense variant | rs709596309 | Productive, fatness and meat quality | 22.868 | [9] |
| PNKP (p.Gln96Arg) | 6:54880241 | Missense variant |  | Lethal recessives | 28.767 | [6] |
| VRTN (g.97614602A>C) | 7:97,614,602 | Noncoding variant | rs709317845 | Vertebrae number | 11.952 | [10] |
| PPARD (p.Gly32Glu) | 7:31281804 | Missense variant | rs80909573 | Ear size, fat metabolism, skin and cartilage development | 21.589 | [11] |
| SYNGR2 (p.Cys63Arg) | 12:3,797,515 | Missense variant | rs3473454700 | Porcine circovirus viral load | 0.00013 | [12] |
| TADA2A (g.38922102G>A) | 12:38922102 | splice donor variant |  | Lethal recessives | 21.848 | [6] |
| MSTN (p.Glu274*) | 15:94,623,834 | Stop gain variant |  | Leg weakness | 38.959 | [13] |
| PRKAG3 (p.Arg250Gln) | 15:120,863,533 | Missense variant | rs1109104772 | Glycogen content and meat quality | 32.657 | [14] |
| PCK1 (p.Met139Leu) | 17:57932233 | Missense variant | rs343196765 | Intramuscular fat content, backfat thickness and meat quality | 23.322 | [15] |

This table was based on published tables for known or proposed causal variants in pigs [16, 17].

**Supplementary table 3.** Comparison of fetal phenotypes grouped by level of log fold change in *DIO2* expression in fetal heart.

|  | Group by log FC of *DIO2* | | |
| --- | --- | --- | --- |
| Fetal genotype and phenotype^¶^ | High^†^ (n=5) | Mid^†^ (n=9) | Mid (n=2) |
| Asn91Ser genotype | CD | CC | CD |
| logddDIO2 | 1.7 (0.4) | -0.2 (0.3) | 0 (0.7) |
| Brain to liver weight | 1.4 (0.1) | 1.3 (0.1) | 1.3 (0.1) |
| **Viral load in placenta** | **5.3 (0.6)** | **3.7 (1.3)** | **1.7 (1.7)** |
| **Viral load in serum** | **7.6 (0.6)** | **4.1 (2.6)** | **1.7 (0.6)** |
| **Viral load in thymus** | **6.8 (0.3)** | **3.5 (2)** | **1.6 (0.7)** |
| **T4 (nmol/L)** | **54.6 (15.5)** | **70.9 (21.9)** | **75.6 (4.1)** |
| T3 (nmol/L) | 0.6 (0.1) | 0.6 (0.2) | 0.6 (0.1) |
| litter size | 17.6 (3.3) | 17.3 (2.3) | 20 (0) |
| Fetal weight (g) | 930.6 (87.6) | 876.6 (126.1) | 932 (39.4) |
| Crown rump length (cm) | 36.9 (1.2) | 35.6 (1.7) | 37.1 (0.1) |
| Girth (cm) | 8.6 (0.4) | 8.4 (0.5) | 8.7 (0) |
| Brain weight (g) | 28.7 (1.3) | 25.9 (2.4) | 29.4 (1.8) |
| Liver weight (g) | 21.2 (2.1) | 20.2 (1.9) | 22.2 (0.8) |
| Heart weight (g) | 6.8 (0.6) | 7.1 (1.2) | 7.8 (0.2) |
| Lung weight (g) | 25.4 (3.2) | 26.4 (4.9) | 28.9 (0.2) |
| Thyroid weight (mg) | 213 (41.9) | 204.9 (55.2) | 243.5 (38.9) |
| Adrenal weight (mg) | 216.8 (49.4) | 146.7 (46.4) | 146.5 (53) |
| Spleen weight (g) | 1.5 (0.2) | 1.3 (0.3) | 1.4 (0.3) |

^¶^Fetal phenotypes were represented by mean (SD); phenotypes were bolded if high group showed a deviation from mid groups.

^†^5 CD fetuses with log fold change (FC) of *DIO2* >1 (High) were compared with 9 CC or 2 CD fetuses with -0.5 ≤ log FC ≤ 0.5 (Mid), while being at the same range of the covariate (1.1 ≤ brain to liver weight ≤ 1.46).

**Supplementary table 4.** Estimated marginal mean (SE or 95% CI) associating Asn91ser genotypes (CC, CD) with fetal outcomes.

|  | Estimated marginal mean (SE or 95% CI) | | | | | |  |
| --- | --- | --- | --- | --- | --- | --- | --- |
| Fetal outcomes | Trial-1 | | | Trial-2 | | | Other predictors included |
|  | CC | CD | *P value* | CC | CD | *P value* |  |
| Fetal viability^†^ | 0.66  (0.41-0.84) | 0.57  (0.32-0.78) | 0.54 | 0.41  (0.22-0.63) | 0.75  (0.54-0.88) | 0.18 | sex |
| Fetal survival^ǂ^ | 0.64  (0.39-0.83) | 0.73  (0.48-0.88) | 0.33 | 0.73  (0.55-0.86) | 0.81  (0.65-0.90) | 0.33 | sex |
| **Viral loads** |  |  |  |  |  |  |  |
| Serum viral loads (log10 copies/ul) | 2.18 (1.36) | 1.58 (1.37) | 0.21 | 5.75 (0.93) | 5.16 (0.93) | 0.21 | fetal preservation, T4 level |
| Thymic viral loads (log10 copies/mg) | 2.48 (1.45) | 1.78 (1.46) | 0.15 | 4.43 (1.00) | 3.73 (0.99) | 0.15 | fetal preservation, T4 level |
| **Thyroid hormone levels** |  |  |  |  |  |  |  |
| T4 in serum (nmol/L) | 25.23 (2.70) | 21.47 (2.86) | 0.16 | 77.11 (2.14) | 73.35 (2.09) | 0.16 | fetal classification, litter size, sex |
| T3 in serum (nmol/L) | 0.59 (0.08) | 0.61 (0.08) | 0.64 | 0.75 (0.06) | 0.77 (0.06) | 0.64 | fetal classification, body weight |
| **Morphometrics** |  |  |  |  |  |  |  |
| Body weight (g)^¶^ | 816.17 (43.60) | 900.73 (44.87) | 0.13 | 996.39 (39.11) | 875.89 (38.75) | 0.13 | fetal classification, litter size |
| Crown rump length (cm)^¶^ | 26.68 (0.60) | 28.15 (0.62) | 0.009 | 37.03 (0.56) | 35.11 (0.56) | 0.031 | fetal classification, litter size |
| **Organ weights** |  |  |  |  |  |  |  |
| Brain (g) | 24.15 (0.60) | 24.13 (0.61) | 0.98 | 26.87 (0.46) | 26.86 (0.46) | 0.98 | fetal classification |
| Ratio: brain to liver weight | 1.08 (0.09) | 1.08 (0.09) | 0.9 | 1.11 (0.07) | 1.10 (0.07) | 0.9 | fetal classification, litter size |

^†^Estimated probability (95% CI) indicated for interaction effect between Asn91Ser and trial, with *P* value adjusted by Benjamini-Hochberg (BH) method across all possible pairwise comparisons.

^ǂ^Estimated probability (95% CI).

^¶^Estimated marginal mean (SE) indicated for interaction effect between Asn91Ser and trial, with *P* value adjusted by Benjamini-Hochberg (BH) method across all possible pairwise comparisons.

**Supplementary table 5.** Determination of sequence variants in pig *TSHR* region for trial-2 sires (N=6).

| position | strand | dbSNP ID | SNP label | sequence change | R^2^.with.Asn91Ser^†^ | *P*.value.LD^ǂ^ |
| --- | --- | --- | --- | --- | --- | --- |
| 103768370 | + | no dbSNP | 5_prime_UTR_variant1 | c.-619T>C | 0.06642523 | 0.37196101 |
| 103768497 | + | no dbSNP | 5_prime_UTR_variant2 | c.-492C>T | 0.06642523 | 0.37196101 |
| 103768500 | + | no dbSNP | 5_prime_UTR_variant3 | c.-489T>C | 0.46621872 | 0.018015671 |
| 103768553 | + | no dbSNP | 5_prime_UTR_variant4 | c.-436C>T | 0.06642523 | 0.37196101 |
| 103768590 | + | no dbSNP | 5_prime_UTR_variant5 | c.-399T>C | 0.46621872 | 0.018015671 |
| 103768716 | + | no dbSNP | 5_prime_UTR_variant6 | c.-273T>C | 0.46621872 | 0.018015671 |
| 103768817 | + | no dbSNP | 5_prime_UTR_variant7 | c.-172A>C | 0.199701409 | 0.206242057 |
| 103768826 | + | no dbSNP | 5_prime_UTR_variant8 | c.-163G>A | 0.110862332 | 0.346319129 |
| 103768890 | + | no dbSNP | 5_prime_UTR_variant9 | c.-99T>G | 0.199701409 | 0.206242057 |
| 103768892 | + | no dbSNP | 5_prime_UTR_variant10 | c.-97_-96insCGAGT | NA^¶^ | NA |
| 103768930 | + | no dbSNP | 5_prime_UTR_variant11 | c.-59A>G | 0.237775307 | 0.09118638 |
| 103847623 | + | rs1112876687 | intron_variant1 | c.171-145G>C | 0.46621872 | 0.018015671 |
| 103847991 | + | rs1111127688 | intron_variant2 | c.242+152C>T | 0.46621872 | 0.018015671 |
| 103853698 | + | rs1107841267 | intron_variant3 | c.317+113_317+114insCT | NA | NA |
| 103867986 | + | rs1108767362 | intron_variant4 | c.318-122G>A | 0.666106717 | 0.004695127 |
| 103868013 | + | rs1109465473 | intron_variant5 | c.318-95A>G | 0.666106717 | 0.004695127 |
| 103868217 | + | No dbSNP | intron_variant6 | c.392+35_392+40insB^#^ | 0.666106717 | 0.004695127 |
| 103874397 | + | rs792895037 | intron_variant7 | c.468-9C>T | 0.030134093 | 0.54761393 |
| 103874498 | + | rs1107939652 | intron_variant8 | c.545+15T>C | 0.110862332 | 0.248743321 |
| 103877467 | + | rs1112574587 | intron_variant9 | c.546-505A>G | 0.46621872 | 0.018015671 |
| 103877514 | + | No dbSNP | intron_variant10 | c.546-458A>G | 0.46621872 | 0.018015671 |
| 103877580 | + | No dbSNP | intron_variant11 | c.546-392C>A | 0.46621872 | 0.018015671 |
| 103877650 | + | rs1110746575 | intron_variant12 | c.546-322G>A | 0.46621872 | 0.018015671 |
| 103877694 | + | rs1112391719 | intron_variant13 | c.546-278T>C | 0.46621872 | 0.018015671 |
| 103877788 | + | rs1113090843 | intron_variant14 | c.546-184A>C | 0.46621872 | 0.018015671 |
| 103877791 | + | rs1110448239 | intron_variant15 | c.546-181G>T | 0.46621872 | 0.018015671 |
| 103877804 | + | rs1111217740 | intron_variant16 | c.546-168T>C | 0.46621872 | 0.018015671 |
| 103877955 | + | rs1110230512 | intron_variant17 | c.546-17A>T | 0.46621872 | 0.018015671 |
| 103889483 | + | rs1109842851 | intron_variant18 | c.615-178A>C | 0.46621872 | 0.018015671 |
| 103923199 | + | rs699096214 | missense_variant | c.1618A>G | NA | NA |
| 103922622 | + | rs196952307 | synonymous_variant1 | p.His347= | 0.46621872 | 0.018015671 |
| 103922631 | + | rs196957158 | synonymous_variant2 | p.Ser350= | 0.46621872 | 0.018015671 |
| 103924033 | + | rs1111686982 | 3_prime_UTR_variant1 | c.*157G>A | 0.46621872 | 0.018015671 |

^†^R^2^.with.Asn91Ser: Linkage disequilibrium (LD) estimate using squared allelic correlation coefficient (R^2^) between the coding variant (p.Asn91Ser) in *DIO2* and each SNP detected.

^¶^NA, LD not estimated since all sires had the same genotype, just differing compared to reference sequence.

^ǂ^*P* value.LD, significance of LD estimate.

^#^B allele denotes inserted alleles collectively compared to reference sequence at SSC7:103868217.

**Supplementary table 6.** Primers used for Sanger sequencing.

| Gene name  (Ensembl Gene stable ID^†^) | Amplicons | Forward primer (5'→3') | Reverse primer (5'→3') | Amplicon length (bp) | Overlap with previous amplicon | Overlap with next amplicon |
| --- | --- | --- | --- | --- | --- | --- |
| DIO2 (ENSSSCG00000040638)^ǂ^ | amplicon_1 | GGCTGGAGAGACTGGACTTG | AGATGGTTCTGCTGCCAACT | 1108 | - | - |
|  | amplicon_2A | CCATGATGGCTCTTTCCTCA | GGGCTCTATCCATGCTGAAG | 1177 | - | 244 |
|  | amplicon_2B | GCTCAAAAGTAGCCCCATCA | GCCTTACATCAAAGCCTCCA | 1150 | 244 | 443 |
|  | amplicon_2C | GAGAATGGCAGATGGAGAGG | TGGAACAAAGGGGAAGTTTG | 1295 | 443 | 404 |
|  | amplicon_2D | TCAGTGTGCAAGAACCAAAAG | ATTCATGCCCATTCAGGAAA | 1268 | 404 | 355 |
|  | amplicon_2E | TTTCAATATCCACCCCACCT | GGGACAGAAGTTGGTGCCTA | 1007 | 355 | 276 |
|  | amplicon_2F | CTGCCTTGCTGCATAAAACA | AGAGCTGCTGCCCAAGATAG | 944 | 276 | 351 |
|  | amplicon_2G | GAACAGGACCTGGGAGATCA | CCAGGGGCAAGTTCTAGAGAG | 1112 | 351 | - |
| TSHR (ENSSSCG00000031771)^¶^ | amplicon_1 | GGCAGACCAGAATTTACCAGTTG | GGCCTCTTCTCAAGCTCTTTTTG | 1124 | - | - |
|  | amplicon_2 | CATCCCAGTCTACTGCTACTTCAG | ACCCGTCGTGAAAAGAGAATCAA | 701 | - | - |
|  | amplicon_3 | AGGACTAATGTAGTGGAGGAACT | GCTGACACGGTAGTACAGAAAG | 813 | - | - |
|  | amplicon_4 | AATCCACTCAAACCTCCACTC | AGTTCCCATCGTGGTTCAATAG | 764 | - | - |
|  | amplicon_5 | CCATGGTCTCTCAGGTAATTAAGG | TTCTTTCTTGAGCCCACTCC | 505 | - | - |
|  | amplicon_6 | TGAAATCCATTGGTCCCATTCT | GACTTTGGGACGGTCACTTAAA | 963 | - | - |
|  | amplicon_7 | CCATGGTCTGTCTGCTTTCT | GGTCGTACTAACTCTGGTGTTG | 759 | - | - |
|  | amplicon_8 | TGCCATTGGCCAGGTAAG | CCAACTATTCACACTCCTCCTTT | 748 | - | - |
|  | amplicon_9 | ACAGGAGTGGGAGCAATTTAG | GCAAGATCTGGTCATCTCCTAAA | 856 | - | - |
|  | amplicon_10 | CTTGAATTGCTTGCAGATGAGAA | GTCTTTGTCTCCCGGGTTATAC | 1198 | - | 418 |
|  | amplicon_11 | GTACTACAACCATGCCATCGAC | GCCATTGTTGCATTTAGCATCT | 1100 | 418 | - |

^†^Ensembl gene ID for Pig genes (Sscrofa11.1) (Ensembl Release 105 (Dec 2021) used).

^ǂ^For *DIO2*, the first coding exon (ENSSSCE00000310588; 275 bp length;) was covered by 1 amplicon (amplicon_1) and the second coding exon (ENSSSCE00000324921; 5666 bp length) covered by 7 amplicons (amplicon 2A to 2G).

^¶^For *TSHR*, a predicted transcript isoform, TSHR-202 (ENSSSCT00000038969.2), was targeted for sequencing. Region of 1^st^ (ENSSSCE00000332089) to 2^nd^ (ENSSSCE00000288966) exon including 1^st^ intron (863 bp length) covered by amplicon_1; translated region (1414 bp length) of 11^th^ exon (ENSSSCE00000324742) covered by 2 amplicons, amplicon_10 and 11. The rest of exons (3^rd^ to 10^th^ exon) covered by each amplicon (amplicon_2 to amplicon_9).

Annealing temperature for all amplicons was 60℃.

**Supplementary table 7.** Primers used for quantitative real-time PCR.

| Gene name  (Ensembl Gene stable ID^†^) | Forward primer (5'→3') | Reverse primer (5'→3') | Annealing temp (°C) | Amplicon length (bp) | |
| --- | --- | --- | --- | --- | --- |
| DIO2 (ENSSSCG00000040638) | CTCGGTCATTCTCCTCAAGC | TCACCTGTTTGTAGGCATCG | 61 | 140 |  |
| ACTB (ENSSSCG00000007585) | CCAGCACGATGAAGATCAAG | AGTCCGCCTAGAAGCATTTG | 60 | 171 |  |
| HMBS (ENSSSCG00000015108) | AGGATGGGCAACTCTACCTG | GATGGTGGCCTGCATAGTCT- | 61 | 83 |  |
| SDHA (ENSSSCG00000020686) | CTACAAGGGGCAGGTTCTGA | AAGACAACGAGGTCCAGGAG | 61 | 141 |  |
| STX5 (ENSSSCG00000026293) | TGCAGAGTCGTCAGAATGGA | CCAGGATTGTCAGCTTCTCC | 60 | 144 |  |

^†^Ensembl gene ID for Pig genes (Sscrofa11.1) (Ensembl Release 105 (Dec 2021) used).

**Supplementary table 8.** Selected fetal population for association analysis.

| Genotyping method | Trials | |
| --- | --- | --- |
|  | trial-1^†^ | trial-2^†^ |
| TaqMan assay | 78 | 59 |
| inferred by parental genotypes from Sanger sequencing | 0 | 86 |
| Total | 78 | 145 |

^†^trial-1, GWAS of Yang et al.(2016) [18]; trial-2, follow up study of Ko et al. (2022) [1].

**References**

1. Ko H, Sammons J, Pasternak JA, Hamonic G, Starrak G, MacPhee DJ, Detmer SE, Plastow GS, Harding JCS. Phenotypic effect of a single nucleotide polymorphism on SSC7 on fetal outcomes in PRRSV-2 infected gilts. Livestock Science. 2022;255:104800.

2. Kim KS, Larsen N, Short T, Plastow G, Rothschild MF. A missense variant of the porcine melanocortin-4 receptor (MC4R) gene is associated with fatness, growth, and feed intake traits. Mammalian Genome. 2000;11(2):131-5.

3. Fontanesi L, Ribani A, Scotti E, Utzeri VJ, Veličković N, Dall'Olio S. Differentiation of meat from European wild boars and domestic pigs using polymorphisms in the MC1R and NR6A1 genes. Meat Science. 2014;98(4):781-4.

4. Van Laere A-S, Nguyen M, Braunschweig M, Nezer C, Collette C, Moreau L, Archibald AL, Haley CS, Buys N, Tally M, Andersson G, Georges M, Andersson L. A regulatory mutation in IGF2 causes a major QTL effect on muscle growth in the pig. Nature. 2003;425(6960):832-6.

5. Ma J, Yang J, Zhou L, Ren J, Liu X, Zhang H, Yang B, Zhang Z, Ma H, Xie X, Xing Y, Guo Y, Huang L. A splice mutation in the PHKG1 gene causes high glycogen content and low meat quality in pig skeletal muscle. Plos Genetics. 2014;10(10):e1004710.

6. Derks MFL, Gjuvsland AB, Bosse M, Lopes MS, Van Son M, Harlizius B, Tan BF, Hamland H, Grindflek E, Groenen MAM, Megens H-J. Loss of function mutations in essential genes cause embryonic lethality in pigs. Plos Genetics. 2019;15(3):e1008055.

7. Fujii J, Otsu K, Zorzato F, De Leon S, Khanna VK, Weiler JE, O'Brien PJ, MacLennan DH. Identification of a mutation in porcine ryanodine receptor associated with malignant hyperthermia. Science. 1991;253(5018):448-51.

8. Otsu K, Khanna VK, Archibald AL, Maclennan DH. Cosegregation of porcine malignant hyperthermia and a probable causal mutation in the skeletal muscle ryanodine receptor gene in backcross families. Genomics. 1991;11(3):744-50.

9. Óvilo C, Fernández A, Fernández AI, Folch JM, Varona L, Benítez R, Nuñez Y, Rodríguez C, Silió L. Hypothalamic expression of porcine leptin receptor (LEPR), neuropeptide Y (NPY), and cocaine- and amphetamine-regulated transcript (CART) genes is influenced by LEPR genotype. Mammalian Genome. 2010;21(11-12):583-91.

10. Fan Y, Xing Y, Zhang Z, Ai H, Ouyang Z, Ouyang J, Yang M, Li P, Chen Y, Gao J, Li L, Huang L, Ren J. A further look at porcine chromosome 7 reveals VRTN variants associated with vertebral number in Chinese and Western pigs. PloS One. 2013;8(4):e62534.

11. Ren J, Duan Y, Qiao R, Yao F, Zhang Z, Yang B, Guo Y, Xiao S, Wei R, Ouyang Z, Ding N, Ai H, Huang L. A missense mutation in PPARD causes a major QTL effect on ear size in pigs. Plos Genetics. 2011;7(5):e1002043.

12. Walker LR, Engle TB, Vu H, Tosky ER, Nonneman DJ, Smith TPL, Borza T, Burkey TE, Plastow GS, Kachman SD, Ciobanu DC. Synaptogyrin-2 influences replication of Porcine circovirus 2. Plos Genetics. 2018;14(10):e1007750.

13. Matika O, Robledo D, Pong-Wong R, Bishop SC, Riggio V, Finlayson H, Lowe NR, Hoste AE, Walling GA, Del-Pozo J, Archibald AL, Woolliams JA, Houston RD. Balancing selection at a premature stop mutation in the myostatin gene underlies a recessive leg weakness syndrome in pigs. Plos Genetics. 2019;15(1):e1007759.

14. Milan D, Jeon J-T, Looft C, Amarger V, Robic A, Thelander M, Rogel-Gaillard C, Paul S, Iannuccelli N, Rask L, Ronne H, Lundström K, Reinsch N, Gellin J, Kalm E, Roy Pascale L, Chardon P, Andersson L. A mutation in PRKAG3 associated with excess glycogen content in pig skeletal muscle. Science. 2000;288(5469):1248-51.

15. Latorre P, Burgos C, Hidalgo J, Varona L, Carrodeguas JA, López-Buesa P. c.A2456C-substitution in Pck1 changes the enzyme kinetic and functional properties modifying fat distribution in pigs. Scientific Reports. 2016;6(1):19617.

16. Groß C, Derks M, Megens H-J, Bosse M, Groenen MAM, Reinders M, De Ridder D. pCADD: SNV prioritisation in *Sus scrofa*. Genetics Selection Evolution. 2020;52(1):1-15.

17. Johnsson M, Jungnickel MK. Evidence for and localization of proposed causative variants in cattle and pig genomes. Genetics Selection Evolution. 2021;53(1):67.

18. Yang T, Wilkinson J, Wang Z, Ladinig A, Harding J, Plastow G. A genome-wide association study of fetal response to type 2 porcine reproductive and respiratory syndrome virus challenge. Scientific Reports. 2016;6(1):20305.
